# Tackling Real-World Environmental Paper Pollution: A Problem-Based Microbiology Lesson About Carbon Assimilation

**DOI:** 10.3389/fmicb.2020.588918

**Published:** 2020-11-05

**Authors:** Jackie E. Shay, Ruth Solis, Marcos E. García-Ojeda

**Affiliations:** Department of Molecular and Cell Biology, School of Natural Sciences, University of California, Merced, Merced, CA, United States

**Keywords:** paper pollution, 5E model, problem-based learning, microbiology, pedagogy

## Abstract

Governmental and educational organizations advocate for the adoption of inquiry-based, student-centered educational strategies in undergraduate STEM curricula. These strategies are known to benefit students by increasing performance, enhancing mastery of class content, and augmenting affect, particularly in underrepresented racial/ethnic minority students. Among these strategies, case study and project-based learning allow students to master course content while collectively tackling relevant, real-world societal problems. In particular, environmental pollution with paper-based products provide a current problem by which microbiology students learn about the role of microorganisms in paper waste management as well as the microbiological and biochemical processes involved in protein secretion, nutrient uptake, and energy metabolism. Delivered in a flipped, hybrid class in a Technology-Enabled Active Learning (TEAL) laboratory, this lesson taught students about exoenzyme secretion, biopolymer hydrolysis, intracellular transport of sugars, and sugar catabolic reactions. Students demonstrated increased comprehension of exoenzyme function and secretion, as well as how cells uptake the products of exoenzyme hydrolysis. However, students had challenges in placing the transported exoenzyme products within metabolic processes. Our results show increased perceived learning from the students as well as an understanding of the societal implications of these microbiological concepts. Our lesson deviated from knowledge silos in which students learn information in discrete topics. While departing from employing traditional, compartmentalized learning approaches, this student-centered guided lesson frames the systemic nature of the microbiological and biochemical processes underlying the decomposition of organic matter in a real-world context.

## Introduction

Institutions and faculty are revolutionizing their science, technology, engineering, and mathematics (STEM) educational programs to effectively engage and train tomorrow’s scientists (AAAS, 2009). Students need more than a traditional biology education to tackle the most pressing issues facing science and society; they need to learn how to address real-world problems. By presenting a problem in an experiential learning environment, students can build their own conceptual framework through an active learning process that encourages them to socially engage, share ideas, and participate in their own inquiry-based learning (Allen and Tanner, 2005; Chorazy and Klinedinst, 2019). Problem-based learning fosters deep, accurate understanding of a subject and contributes to developing process skills such as research, teamwork, and verbal communication (Allen et al., 2011; Orsmond and Zvauya, 2015). Problem-based learning increases awareness and connects students to local challenges, which is instrumental in actively engaging them in their education, especially for underrepresented student populations (Nelson-Hurwitz and Buchthal, 2019).

When students are passionate about a subject, they engage deeply in their learning (Harackiewicz et al., 2016), and one issue that students are passionate about is the environment (Desa et al., 2011). A growing global crisis is waste pollution, and contrary to many perspectives, paper is a major contributing factor to global waste. The average American consumes 700 pounds of paper each year and paper represents 25% of waste in landfills and 33% of municipal waste (Desilver, 2016). Now more than ever, it is important to foster students who can critically assess these issues by understanding how natural biodegradation cycles operate to ameliorate stress on our global ecosystem (Marope, 2016). The use of deliberate pedagogy can increase awareness of paper waste pollution and emphasize how microbial degradation provides an alternative to paper waste management (Nelson-Hurwitz and Buchthal, 2019).

In this paper, we test the use of evidence-based teaching practices in combination with problem-based learning in a flipped classroom to ask if these have a positive impact on students’ understanding of complex biological material in the context of a real-world problem. Specifically, we wanted to ascertain if the activity would increase student’s mastery of the concepts of bacterial protein secretion, nutrient transport, and carbon cycle. Furthermore, we wanted to valuate if the activity would influence the student’s confidence about their knowledge of these topics. Here we present an effective lesson that utilizes paper waste degradation as a platform to teach students about the carbon cycle. Our work shows that this lesson increases student’s awareness of paper waste pollution and management, content knowledge about microbial paper degradation, and mastery of the process of bacterial protein secretion, nutrient transport and metabolism.

### Pedagogical Framework and Principles

#### The Technology-Enabled Active Learning (TEAL) Laboratory Environment

The General Microbiology course at the University of California, Merced (UC Merced) is a hybrid class taught using flipped pedagogy, and it is delivered in a TEAL laboratory environment. The TEAL lab is a classroom space designed to offer students the opportunity to enhance their cognitive and behavioral engagement through small group discussions, peer evaluation, and shared experiential learning. Students interact more with each other, share resources, and experience a more equitable learning environment in the TEAL lab (Beichner et al., 2007). These labs facilitate the implementation of active learning strategies to best utilize the classroom space (Cotner et al., 2013).

The General Microbiology TEAL lab houses 90 students in ten working tables (Supplementary Figure 1). These are arranged to allow equal view of two large class projector screens located in the front and back of the room. Each table is equipped with docks to power laptops, a document camera to display paper-based work, a whiteboard, an HDMI monitor and a control panel. The instructor control lectern is centered in the room and has the ability to orchestrate the technology offered in the space. In this way, class content can stream from the lectern, or from any of the ten working tables to the entire class (Office of Information Technology, UC Merced, 2020).

#### Active Learning and Flipped Hybrid Classrooms

Active learning is a student-centered pedagogy where students interact with their learning as opposed to passively listening to a lecture. It is widely accepted for its efficacy in increasing students’ performance, especially in STEM courses (Freeman et al., 2014). In order to use the space and time with the students most effectively, we developed a flipped classroom model designed to center the class around the students through facilitated experiential learning. Students watch class content videos at home and attend their scheduled lecture hour prepared for activities with a heightened sense of engagement (Gilboy et al., 2015). The hybrid flipped classroom provides students with a more intimate experience with their instructors, where they can benefit from team-based learning, demonstrate their knowledge, and receive immediate feedback.

To amplify the effects of an active team-based learning environment, our lesson structure follows a recommended instructional protocol by Michaelsen and Sweet (2011). Students begin class with readiness assurance practices, where they (1) watch video lectures before class; (2) respond individually to a 10-min lecture comprehension quiz at the start of lecture (Supplementary Material 2); (3) review the quiz as a table to confirm answers; and (4) discuss the quiz results as a class to identify and diffuse misconceptions and establish consensus. The readiness assurance is followed by a 55-min flipped lecture, which consists of a brief review of material followed by an activity based on the 5E model.

#### The 5E Model: Engaging Large Classes

To address the needs of a large active-learning class, it is necessary to implement a well-documented form of learning that focuses on the desired learning outcomes and considers the cognitive engagement of the students. The learning-cycle method known as the “5E model” (Bybee, 1997) is a common method for organizing large biology-based lessons infused with active learning (Allen and Tanner, 2005). The 5E model, comprised of five stages (engage, explore, explain, elaborate, and evaluate), is known for its efficiency and positive effects on students’ broad-scale academic achievement, attitudes toward lessons, and science process skills (Cakır, 2017).

In order to bring the 5E framework into the context of our microbiology lesson, we integrated a constructivist approach to help students assemble their own knowledge and build a cooperative teamwork dynamic (von Glasersfeld, 1987; AAAS, 2009; Arik and Yilmaz, 2020). We used backward design to create class materials, first defining the learning outcomes and then charting steps to reach the desired level of understanding (Wiggins and McTighe, 1998). We developed activities, discussions and questions that would help students accomplish those outcomes. We integrated a team-based learning experience, proven to foster authentic perspectives to complex problems beyond the scale of individual learning (Michaelsen and Sweet, 2011). This established team-based approach, together with active-learning, helps underrepresented students succeed in STEM courses (Freeman et al., 2014; Snyder et al., 2016). This is particularly important at UC Merced, a Hispanic Serving Institution with over 70% first generation students.

## Materials and Methods

### Research Setting

#### The University of California, Merced

UC Merced is the first research university built in the 21st century in the United States and serves the predominantly underserved communities of California’s San Joaquin Valley. UC Merced holds a diverse student population, with 87% of students from traditionally underrepresented groups. The university has over 8,800 students and is designated as both a Hispanic Serving Institution (HSI) and an Asian American/Native American/Pacific Islander Serving Institution (AANAPISI).

#### Participants

Our student population included 89 students in the Fall 2019 course and 90 students in the Spring 2020 course (*n* = 179). The predominant racial and ethnic make-up of the combined cohorts consisted of 34.1% Asian and 33.5% Hispanic/Latinx, reflecting the University’s HSI and AANAPISI designations. The remaining student population in the course was comprised of 2.8% American Indian/Alaska Native, 5% Black, 3.4% Native Hawaiian/Pacific Islander and 15.6% White. The cohort is primarily comprised of female students (65.4%). The mean age is 21 years of age. The primary difference between the cohorts is class level; the Fall 2019 class was mostly Senior students (93%) while the Spring 2020 cohort had 31% Juniors and 69% Seniors. Students GPA did not differ significantly between the two cohorts [*t*(177) = −1.22, *p* = 0.22], being 2.89 for Fall 2019 and 2.96 for the Spring 2020 (Table 1).

**TABLE 1:** Student participant demographics.

	Fall 2019		Spring 2020		Overall	
	*n* = 89		*n* = 90		*n* = 179	
	*n*	(%)	*n*	(%)	*n*	(%)
**Gender**						
Male	26	(29.2)	36	(40.0)	62	(34.6)
Female	63	(70.8)	54	(60.0)	117	(65.4)
**Race/ethnicity**						
American Indian/Alaska Native	2	(2.2)	3	(3.3)	5	(2.8)
Asian	28	(31.5)	33	(36.7)	61	(34.1)
Black	5	(5.6)	4	(4.4)	9	(5.0)
Hispanic/Latinx	32	(36.0)	28	(31.1)	60	(33.5)
NH/PI^1^	3	(3.4)	3	(3.3)	6	(3.4)
White	14	(15.7)	14	(15.6)	28	(15.6)
Two or more races	3	(3.4)	4	(4.4)	7	(3.9)
Declined^2^	2	(2.2)	1	(1.1)	3	(1.7)
Age — mean (range)	21.3 (18–23)		21.3 (20–27)			
**Class — (%)**						
Junior	(7)		(31)			
Senior	(93)		(69)			
GPA^3^ — mean ± *SD*	2.89 ± 0.38		2.96 ± 0.42		2.92 ± 0.41	

*^1^NH/PI, Native Hawaiian/Pacific Islander. ^2^Students who declined to report race/ethnicity. ^3^Grade Point Average.*

#### Format of General Microbiology at UC Merced

General Microbiology (BIO120) is an upper division course taken primarily by biology majors, and it is a graduation requirement for students in the Microbiology and Immunology emphasis track. The course requirements include General Biology, Molecular Biology, and Cellular Biology. The most relevant content to General Microbiology that students learn in these pre-requisite courses includes the overall organization and structure of cells, principles of metabolism and nutrient cycles, as well as the function of enzymes.

General Microbiology meets twice a week for 75 min in the TEAL lab, limiting the class size to 90 learners. The teaching team includes the instructor of record (García-Ojeda), one co-instructor (Shay), and one undergraduate learning assistant (Solis). This course was transformed into a flipped, team-based learning class in Fall 2015 and converted into a hybrid course in Fall 2019. The hybrid model used in this class consist of in-person flipped lectures with online discussion sections (Supplementary Material 5). The academic year 2019–2020 was the first full year to incorporate all learning and teaching techniques discussed in this manuscript.

### Learning Outcomes

We chose the global issue of environmental pollution by paper-based products to teach students about the role of microorganisms in paper waste management. At this point in the course, students have been introduced to the history and fundamentals of microbiology, the structures and organization of microbial cells, the evolution and diversity of microbes, microbial motility, microbial growth, nutrient transport, and the nitrogen cycle. This section of the class focuses on carbon acquisition as well as how microbes secrete exoenzymes to hydrolyze macromolecules. Specifically, students learn about the process of bacterial protein secretion, and how different exoenzymes break down lipids, nucleic acids, proteins, and carbohydrates. Students are then asked to connect the process of active nutrient transport via symporters, ABC transporters, or phosphoenolpyruvate-carbohydrate phosphotransferase system (PTS). Lastly, students demonstrate how the transported molecules are further modified by converting enzymes and then metabolized.

#### General Microbiology Course Learning Outcomes

The General Microbiology Course Learning Outcomes were designed to integrate previous learning and develop advanced scientific skills such as research and critical thinking. The following course learning outcomes related to this activity are:

(1) Recognize microbiological concepts and terms used in the primary scientific literature and to communicate with other microbiologists and scientists.
(2) Discern the molecular, metabolic, structural, and ecological differences between microbial cells and be able to explain how these differences allow microbes to (a) live in almost any environment on earth, (b) sense, react, and interact with their environment as well as with other organisms, and (c) serve as tools for science, medicine, and industry.
(3) Synthesize knowledge gained in previous courses and apply it to novel microbiological questions.

#### Carbon Acquisition Lesson Learning Outcomes

This lesson has the following learning outcomes:

(1) Given the biotic and abiotic sources of carbon and carbon-containing compounds, illustrate the biological flow of carbon, starting from an initial, complex carbon-containing molecule to a final product (CO_2_ or fermentation product).
(2) Illustrate the enzymatic reactions that hydrolyze carbohydrates, nucleic acids, lipids, and proteins, and identify the various exoenzymes involved in this process.
(3) Identify how different microbes secrete important proteins.

For this lesson, we focused on the first two outcomes by asking students to work with a complex carbon-containing product: paper. Students work through the process of carbon flow, from the initial biomolecule carbon source in paper (cellulose) to the final metabolic product (CO_2_ or a fermentation product) within Gram-negative bacterial cells. Students build an understanding of the overall carbon cycle and how biomolecules are hydrolyzed by exoenzymes secreted by bacteria. Exoenzyme secretion reinforces learning outcome 3, where students learn about the Sec and Tat translocators as well as the Type V secretion system and related proteins. Students also connect this lesson to previously taught material concerning the transport of monomeric sugar molecules across the cell membrane via PTS transporters as well as the catabolic reactions needed to extract energy under aerobic or anaerobic conditions (glycolysis, Entner-Doudoroff Pathway and Krebs cycle). Lastly, this lesson reinforces previously learned concepts of bacterial cell wall structure, proteins, cell membrane and their functions.

### Pedagogical Format

#### Combining Strategies: Carbon Assimilation Group Exercise Using the 5E Model

The novelty of this pedagogical approach is the combination of strategies which seamlessly merge our highly active and collaborative learning initiatives. For this particular 75-min flipped lesson, students prepare by watching online videos about the carbon cycle, protein secretion systems, exoenzymes, and converting enzymes (for slides of this lecture, see Supplementary Material 1.1). They are also encouraged to listen to a podcast titled “How much of our stuff actually gets recycled?” (Brand, 2018).

A detailed summary of the lesson’s timeframe can be found in Supplementary Table 1. For the *engage* phase of the 5E model, students spend the first 8 min of class taking a readiness assurance quiz (Supplementary Material 2). The quiz is followed by a short discussion of the lesson’s learning outcomes (2 min) and a 5-min in-class lecture where students are introduced to current recycling challenges resulting from the adoption of *National Sword*, a 2018 recycling policy instituted by China, as well as a similar policy by India (Staub, 2020), that banned the import of most recycled materials and set strict contamination limits on recyclables (Allan, 2018). These nations used to purchase the majority of recycled paper from the United States (Hamel, 2018), which now piles up in landfills. To maintain their engagement and bring home the effects of this policy, the class is asked “*What type of paper products would be rejected under China’s National Sword policy?*” After a 5-min discussion, students reply that most paper products contaminated with food, such as pizza boxes, soiled newspaper and paper towels would be rejected. They further conclude that these paper products would end up in a landfill (for slides used during this flipped lesson, see Supplementary Material 1.2).

This introduction is followed by a short 5-min lecture on how cardboard and paper products originate from the processing of plants and trees. A figure from Bayer et al. (2007) is used to illustrate the fate of plant material processed by the paper industry, which generates paper products for human consumption. Once used, these products end up in municipal solid waste facilities where some are recycled, composted, or incinerated. The large majority end up in landfills becoming environmental pollution (Bayer et al., 2007).

**FIGURE 1:**
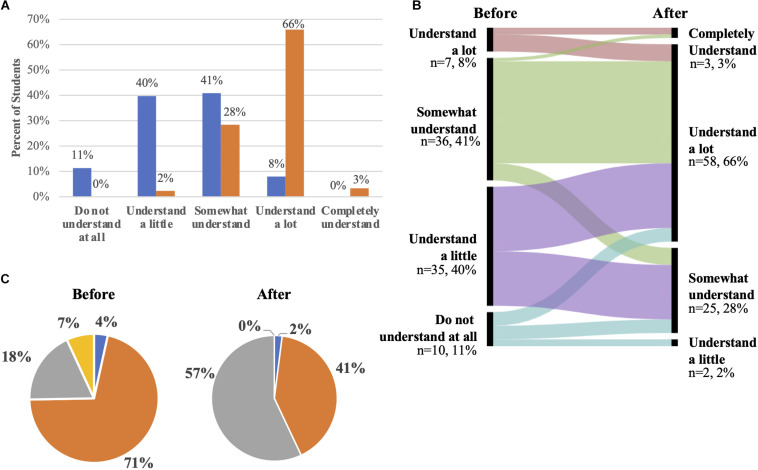
Comparison of student’s responses to metacognitive questions before and after the activity. **(A)** Students were asked the question *“To what extent did you understand the role played by microbes in the biodegradation of paper waste products?”* before and after the class activity. The graph displays the responses before the activity (blue bars) and after the activity (orange bars) for the Spring 2020 semester (*n* = 88). This 5-point Likert survey question was deployed via clickers before the activity or via the Learning Management System after the class. Numbers above the columns represent the percent of students selecting a response. **(B)** Alluvial plot mapping the change in prediction before and after the activity from students in Spring 2020. The left column indicates the predictions before the activity, while the right column indicates the predictions after the activity. Only statements included in the data are shown. **(C)** Pie chart showing student’s predictions of the fate of paper before and after the activity. After a brief introduction, students were asked to predict the fate of paper in a landfill via a multiple-choice clicker question. The answer choices included “*It remains in the landfill, as paper is not degradable.*” (blue), “*It will decompose by microbial activity involving respiration.*” (orange), “*It will decompose by microbial activity involving fermentation.*” (gray), and “*Something else will happen.*” (yellow). The graphs display the before (left pie) and after (right pie) responses for the Spring 2020 semesters (*n* = 88).

Before they reflect on the material, students spend a few minutes answering a metacognitive survey question *“Before today, to what extent did you understand the role played by microbes in the biodegradation of paper waste products?”* via clickers (Supplementary Material 1.2). Following this, students are asked to predict what happens to paper in a landfill, selecting one of the following options in a clicker question: *“It remains in the landfill, as paper is not degradable,” “It will decompose by microbial activity involving respiration,” “It will decompose by microbial activity involving fermentation,”* and *“Something else will happen.”* Together, these questions provide a baseline assessment of the students’ understanding of the role played by microorganisms in the biodegradation of paper waste products (Figure 1C and Supplementary Figure 2A).

For the *explore* phase, students spend 10 min researching and reflecting on different aspects of the molecular composition of paper, the microbial communities that degrade paper, the process of exoenzyme secretion, cellulose hydrolysis, and glucose transport and discuss their findings with their table mates. Each student group is divided into 2 subgroups, and each subgroup is tasked with providing the answer to 3 questions (Supplementary Material 1.2). One subgroup researches the answers to the following questions: *“What is the molecular composition of cardboard and paper?”, “Which microorganisms would degrade cardboard and paper?”, “Are these biochemical processes happening aerobically or anaerobically?”*, and *“Which exoenzymes would these organisms use?”*. The second subgroup researches the questions: *“How would these exoenzymes be secreted?”, “How would the products of the exoenzymatic reactions be transported into the cytoplasm of the bacteria?”*, and *“Once in the cytoplasm, what biochemical processes would be used in catabolic reactions?”*. For the *explain* phase, the instructors help students synthesize their new knowledge and clarify misconceptions during a 10-min class discussion where students from different tables discuss the answers to these questions as a whole class.

**FIGURE 2:**
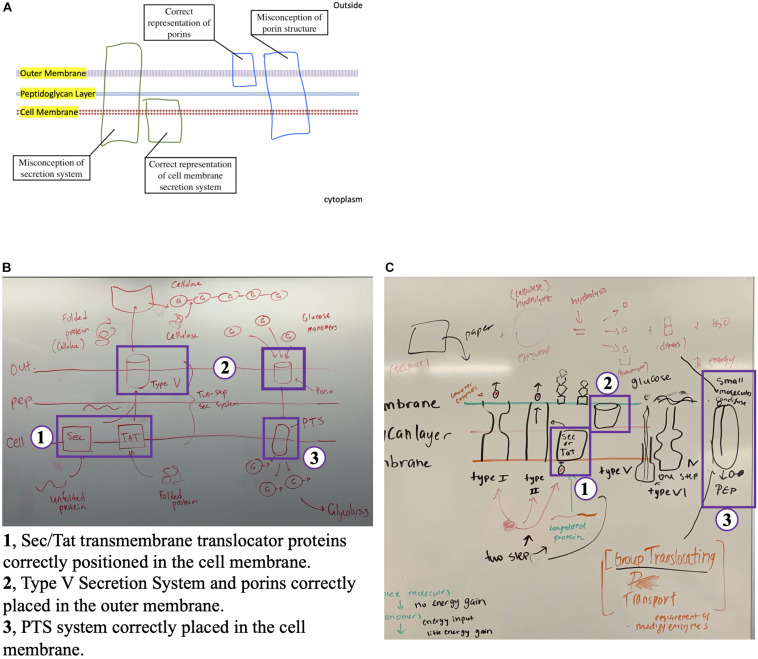
Illustration of enzyme secretion, macromolecule degradation and nutrient transport. Individual groups of students were provided a template to illustrate the processes of enzyme secretion, cellulose hydrolysis and glucose transport. **(A)** Provided template containing the cell envelope of a Gram-negative bacteria, illustrating the correct placement of cell membrane secretion systems and porins, as well as the common misconceptions illustrated by students. **(B)** Correct illustration and **(C)** illustration with misconceptions. The numbers in these images, and the legend, show the most common misconceptions. PTS, phosphotransferase system; Tat, twin-arginine translocation.

In the second part of the activity, the *elaborate* phase, students spend 15 min applying their knowledge by drawing the entire paper-degradation process in their table groups. The drawing must include the main components of exoenzyme secretion, the enzymatic hydrolysis of cellulose happening outside the cell, the transport of glucose into the cell, and finishing with glycolysis (Figure 2). After discussing their drawings, students are again asked to predict what happens to paper in a landfill, and their responses are recorded using clickers (Figure 1C and Supplementary Figure 2B). Finally, the class spends the last few minutes discussing their predictions and editing their figures to present their final drawing online.

#### Data Acquisition, Analysis, and Statistics

Students are *evaluated* at multiple points. Formative assessments include metacognitive questions before, during and after class via clickers or the LMS, drawing their images as well as re-drawing them after in-class discussion. Summative assessments include the readiness assurance quiz and midterm exam questions related to the topic (Supplementary Materials 2, 3).

Students in the Spring 2020 semester were given a post-class metacognitive survey via the LMS where they were asked the following metacognitive questions:

(1) What was the most confusing concept in today’s class?
(2) Based on today’s work, tell us what you think about the following statement: the power of microbes can be harnessed to reach environmentally sustainable goals.
(3) Does today’s work illustrate the relationship of microbiology to society? Explain.
(4) Tell us how much you agree with this statement: Today’s activity did NOT influence my opinion about recycling paper waste.
(5) Tell us how much you agree with this statement: After today’s activity, I will be able to explain how enzyme secretion and sugar transport are related to cell wall and membrane function.

Students in the Fall 2019 cohort only answered the first question. All statistical analyses were conducted in Microsoft Excel version 16.37.

##### Generation of a codebook

Students responses to the metacognitive question *“What was the most confusing concept in today’s class?”* were analyzed for emergent themes in potential missing knowledge, misinterpretation, and misconceptions of class content, following the process described by Offerdahl and Montplaisir (2014). Briefly, two coders (García-Ojeda and Solis) independently read 20 randomly selected student responses (10 from each cohort) for emerging patterns and established an initial code. This initial code was used to code all 157 submissions (From Fall 2019 and Spring 2020 cohorts). The coders then compared and discussed the initial codes for each independent submission, exploring discrepancies in detail, adding new codes, and collapsing or eliminating other codes. This process was done twice more, using the entire sample, to verify and generate a final codebook (Supplementary Material 4). Codes that appeared at least 3 times were kept in the final codebook, consisting of 12 codes in 3 categories. After these discussions, both coders recoded the entire sample. Cohen’s kappa values were calculated to determine intercoder reliability (Dewey, 1983; Glen, 2014) using the following equation

$$\kappa =\frac{p_{\text{o}}-p_{\text{e}}}{1-p_{\text{e}}}$$

where *p*_*o*_ is the relative agreement between both coders, and *p*_*e*_ is the hypothetical probability that agreement was achieved by chance. Kappa values between 0.61–0.8 are considered substantial agreement, while values between 0.81–0.99 are considered near perfect agreement (Landis and Koch, 1977).

Jaccard coefficients have been used to establish the level of consistency between coders (Smith et al., 2013). We calculated the Jaccard coefficient *T* using the equation *T* = *n*_c_/(*n*_a_ + *n*_b_−*n*_c_), where *n*_*c*_ is the number of times a statement was coded the same by both coders, *n*_*a*_ is the number of times a statement was coded the same by both coders plus the times it was coded by coder 1 but not coder 2, while *n*_*b*_ is the number of times a statement was coded the same by both coders plus the times it was coded by coder 2 but not coder 1. Jaccard coefficients closer to 1 are indicative of high consistency between coders (Niwattanakul et al., 2013).

##### Word cloud analysis

Word clouds can be used to investigate patters in text data (DePaolo and Wilkinson, 2014). For metacognitive questions 2 and 3 (see “Data Acquisition, Analysis, and Statistics” section for Metacognitive Topic Questions), we identified and ranked key topics emergent in the students’ answers by using the online software wordclouds.com.^1^ Briefly, we transferred the text responses from the LMS to Microsoft Word and identified and deleted words and phrases that were directly taken from the questions. Such language included phrases like “the power of microbes” or “the relationship of microbiology to society.” We also expunged phrases like “I agree because”. We then uploaded these .docx files into the wordclouds.com website to create the initial word clouds and produce a term table with the term’s respective weights, shown in parenthesis (#, Supplementary Table 2).

Using the term tables, we narrowed down the number of terms in the word cloud by identifying terms that had similar iterations or meanings and adding their respective weights. For example, the terms Microbes (11), microbes (61) and microbe’s (2) were collapsed into the term microbes (74). Words that had meanings unrelated to the question were also eliminated, such as “yes,” “yet,” “terms,” etc. This process reduced the number of terms in the Power of Microbes word cloud from 575 to 126, while reducing the number of terms in the Microbes and Society word cloud from 722 to 248. To reduce the complexity of the word cloud images, we did not include in the illustration terms that appeared less that 4 times.

### Adapting to the COVID-19 Pandemic

The COVID-19 pandemic escalated a few weeks into the Spring 2020 semester and university campuses worldwide shut down to prevent the spread of the SARS-CoV2 virus (Richardson, 2020). Since this course is a flipped, hybrid class, transitioning to ERI required minor adjustments. Although the activities described here were deployed in-person before transitioning to ERI, the exam evaluating the content of this activity was administered online. The Spring 2020 students had to navigate the transition period only a week before they took the exam discussed in this paper.

To deter cheating, the exam was open book, open note, timed and delivered via the LMS using Respondus Lockdown Browser^®^ without the Respondus Monitor^®^. The exam questions were written to build upon foundational knowledge, which require students to use higher order thinking to answer them correctly. Therefore, the answers to these questions could not be easily found online. To clarify exam misconceptions and answer students’ questions, the instructors were available via Zoom during the entire exam period.

## Results

### Formative Assessments

#### Role of Microorganisms in Paper Degradation

During their pre-class metacognitive survey, we asked students to evaluate their understanding of the role of microbes in paper biodegradation. The majority of students reported improved understanding after the activity (Figure 1). Before the activity, over half of students (51%) reported “do not understand at all” or “understand a little,” while 41% reported “somewhat understand” and 8% reported “understand a lot” (Figure 1A, blue bars). After the activity, the vast majority of students reported, “understand a lot” (66%) or “completely understand” (3%) while only 28% reported “somewhat understand” and few (2%) reported “understand a little” (Figure 1A, orange bars).

Using alluvial plots (Figure 1B), we examined the changes in reported understanding of the material by comparing their responses at baseline and after the lesson. Most students reported improvement in their understanding of the material. Some students who initially reported “do not understand at all” (Figure 1B, light blue) reported “understand a little” after the lesson, while the majority of students in this baseline response reported either “somewhat understand” or “understand a lot” after the lesson. The group of students who initially answered, “understand a little” (Figure 1B, lilac), split equally to reporting “somewhat understand” and “understand a lot” after the lesson. The students initially reporting “somewhat understand” (Figure 1B, light green) reported “understand a lot” after the lesson. From students (8%) who reported “understand a lot” at baseline (Figure 1B, rose), subsequently reported “completely understand” and “understand a lot” after the lesson. Taken together, our data indicate that students perceived gains in their understanding of the role of microbes in paper biodegradation after completing the lesson.

#### Mechanism of Paper Biodegradation

Before starting the activity, students were asked to predict the fate of paper in a landfill via a multiple-choice clicker question. Paper buried in a landfill would be degraded primarily by anaerobic mechanisms (Pommier et al., 2010). Initially, the majority of students in the Spring 2020 semester (71%) predicted that respiration played a role in paper biodegradation in landfills (Figure 1C). About 18% stated that paper would be degraded via fermentative pathways, and 11% predicted that something else would happen (Figure 1C). When asked the same question after the activity, students in the Spring 2020 cohort changed their prediction. Only 41% predicted that paper would be degraded via respiratory mechanisms, while the great majority (57%) stated that fermentation would be the primary pathway of paper degradation (Figure 1C). Fewer students (2%) were unsure about the fate of paper in landfills after the activity than before. The greatest transition in answers shifted from students predicting that respiration would play a role to predicting that fermentation would play a role [Supplementary Figure 2B (light green)]. Taken together, the majority of students correctly predicted that paper would be degraded anaerobically via fermentative processes after participating in the lesson.

#### Active Learning Group Exercise: Drawing the Enzyme Secretion, Macromolecule Degradation and Nutrient Transport Pathways

Students demonstrated their overall understanding of class material in a group formative assessment, where they drew the processes of enzyme secretion, cellulose degradation, glucose transport and glucose metabolism (Figure 2). This activity was designed to evaluate the extent by which groups of students engaged in model-based reasoning by using a drawing-to-learn approach (Quillin and Thomas, 2015). A group with high mastery of the material would place the Sec/Tat protein secretion systems across the cell membrane, the Type V Secretion System and the porin channels in the outer membrane, and the glucose PTS across the cell membrane (compare Figures 2B,C). Their drawings will also show the phosphorylation of glucose to glucose-6-phosphate after transport via the PTS system, leading directly to its hydrolysis via glycolysis. Moreover, they would illustrate cellulose as a polymer as well as have appropriate labels for the cellulase enzyme and other components.

#### Metacognitive Topic Questions

##### Student’s identified the topics most confusing to them

Question 1: What was the most confusing concept in today’s class?

To evaluate this question, we established and validated a 12-item codebook (Supplementary Material 4) to explore emerging patterns in students’ responses. These codes were subdivided into 3 categories: (1) Concepts, (2) Competencies, and (3) Affect. Table 2 shows the frequency (*f*) of these codes for each semester, as well as the Jaccard coefficient (*T*), and Cohen’s kappa (κ ± SE) values evaluating inter-rater reliability. The high Jaccard coefficient (≥0.92) and Cohen’s kappa values (≥0.81) indicate a high level of consistency between the two coders.

**TABLE 2:** Codes used to evaluate responses to the question: “*What was the most confusing concept in today’s class?”*.

Category^1^	Code	*T*^2^	κ^3^	*SE* (κ)	*f_*Fall*__19_*	*f_*Spring*__20_*	*f*_*Total*_^4^
Concepts	Biochemistry	0.99	0.96	0.04	10	4	14
	Exoenzyme activity	0.99	0.89	0.07	5	4	9
	Location	1.00	1.00	0.00	3	2	5
	Misconception	0.99	0.95	0.04	16	6	22
	Nutrient transport	0.97	0.82	0.09	4	6	10
	Protein export	0.98	0.95	0.03	19	27	46
	Other	0.99	0.85	0.15	2	1	3
Competencies	Big picture	0.92	0.81	0.05	14	25	39
	Illustration	0.97	0.89	0.05	12	8	20
	Time	1.00	1.00	0.00	1	2	3
Affect	Concern	0.99	0.89	0.11	2	2	4
	Improvement	0.99	0.96	0.03	12	15	27

*^1^Categories: Concepts refer to codes that address students’ questions about class content. Competencies refer to codes describing skills students master throughout the course. Affect refers to codes associated with student’s feelings. ^2^Jaccard coefficient (T) and ^3^Cohen’s kappa (κ) values were calculated with the combined Fall 2019 and Spring 2020 data (n = 157). ^4^Frequency (f) indicates the number of times a code, selected by both coders, appeared in student’s responses. Fall 2019 (n = 72), Spring 2020 (n = 85).*

The Concepts category includes codes that deal with students’ questions about class content. In this category, students from both semesters primarily identified Protein Export as the most confusing concept. This code was used 19 times by Fall 2019 students and 27 times by Spring 2020 students. Biochemistry and Misconceptions were more represented in the Fall 2019 cohort compared to the Spring 2020 cohort. To a lesser degree, but with similar frequencies, both cohorts identified Exoenzyme Activity, Location, and Nutrient Transport as confusing topics.

The Competencies category includes codes that describe skills students ought to master over the course of the lesson. Under this category, Big Picture was the most common code (overall frequency of 39 times), found more frequently in the Spring 2020 cohort. Illustration was also frequently cited, (20 times) with the Fall 2019 cohort displaying this code at a higher frequency. Some students in both cohorts reported having challenges with Time to complete the activities (3 times total).

Although not prompted by the question, students from both cohorts expressed ideas about feelings of self-improvement in conceptual understanding or skills. Some students also expressed feelings of concern about their understanding or feared potential negative consequences for not understanding the material. To reflect the frequency of these statements regarding feelings, we created a category of Affect (Table 2). Improvement was the most frequently used code in this category, with both cohorts displaying similar frequencies for this code. Similarly, Concern was also equally represented to a lesser degree in both cohorts.

##### The power of microbes and the relationship of microbiology to society

Question 2: Based on today’s work, tell us what you think about the following statement: the power of microbes can be harnessed to reach environmentally sustainable goals.

**FIGURE 3:**
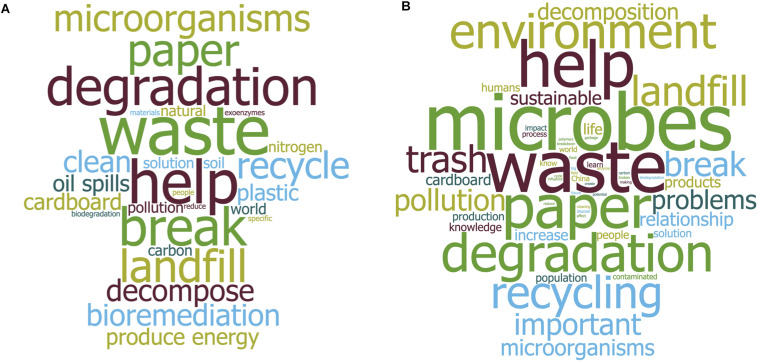
Word clouds illustrating terms in the responses to metacognitive questions. **(A)** Microbes and environmental goals. Following the activity, students were asked the question: *Based on today’s work, tell us what you think about the following statement: the power of microbes can be harnessed to reach environmentally sustainable goals.* **(B)** The relationship between microbiology and society. Following the activity, students answer the question: *Does today’s work illustrates the relationship of microbiology to society? Explain.* Both of these free-response survey questions were deployed via the Learning Management System after the class. Text from the student’s responses was organized using a word cloud software that highlights, by word size, how often a term was used. The images display the responses from the Spring 2020 semester (*n* = 88). To simplify the image, only terms used at least 4 times are shown.

Concerning the power of microbes to reach environmentally sustainable goals, the 10 most used terms (in order of weight) included waste, help, degradation, break, paper, landfill, recycle, clean, decompose and microorganisms (Figure 3A and Supplementary Table 2). The frequency of use for these terms indicates that students perceived microorganisms as a clean alternative to help the degradation (break or decompose) of waste in landfills. The following student responses were selected because they represent the breadth of perspectives and backgrounds of the students:

“…Microbes are definitely beneficial for sustainability … One example can be like bioremediation where microorganisms aid in cleaning air, soil, and water.”

“…We could potentially use bacteria to degrade cardboard that does not fall under conditions to be recycled by parties in other countries. We would be able to continue working on sustainability efforts of decreasing soiled cardboard and paper pollution.”

“…In the lecture prep videos we were able to see the importance of degradation of oil and petroleum by bacteria using oxygenase for bioremediation.”

##### Students understand the connection to of this microbiology lesson to society

Question 3: Does today’s work illustrate the relationship of microbiology to society?

Concerning the relationship of microbiology to society, students connected the activity to four overall societal applications, (1) waste management, (2) sustainability and environmental solutions to pollution, (3) biodegradation as a tool, and (4) energy cycles essential for human life (Figure 3B and Supplementary Table 2). In particular, students reported that the activity helped them understand the real-world implications of the biology they were learning:

“… Listening to the newscast about the garbage problem with China really made it apparent to me how important it is for us in America to find a reliable, safe, and sustainable solution to the ever-increasing garbage problem. I see how microbes can be used to help break down certain types of waste and I am very curious to see if there will be newer research and findings regarding microbe usage in the waste management sector.”

“The activity helped me to connect everything together especially by relating it to the current problem we have with paper not being properly recycled. The activity helped me envision how paper was broken down via cellulase enzyme then brought in by porins and PTS systems and through catabolic reactions we harvested energy. The activity made everything full circle for me.”

##### Students agree: the lesson influences their opinion about paper recycling

Question 4: Tell us how much you agree with this statement: Today’s activity did NOT influence my opinion about recycling paper waste.

**FIGURE 4:**
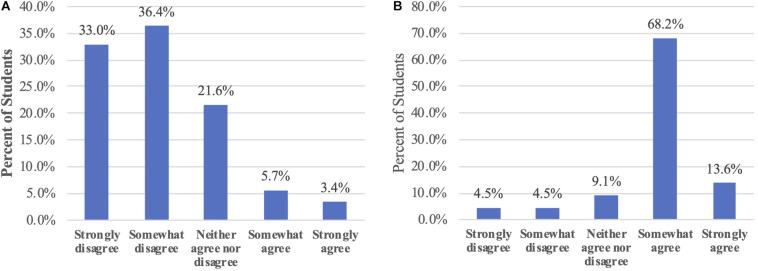
**(A)** The activity strongly influences student’s opinions about recycling. Following the activity, students were asked: “*Tell us how much you agree with this statement: Today’s activity did NOT influence my opinion about recycling paper waste.”* **(B)** The activity strongly enhances student’s understanding of enzyme secretion and nutrient transport. Following the activity, students were asked: “*Tell us how much you agree with this statement: After today’s activity, I will be able to explain how enzyme secretion and sugar transport are related to cell wall and membrane function.”* These 5-point Likert survey questions were deployed via the Learning Management System after the class. The graphs display the responses from the Spring 2020 semester (*n* = 88). Numbers above the columns represent the percent of students selecting a response.

To evaluate if the lesson influenced the students’ opinion about paper recycling, we deployed the above 5-point Likert scale question via LMS. Most students either Strongly disagreed (33%) or Somewhat disagreed (36%) that the activity did not influenced their opinion about recycling paper waste (Figure 4A). About a fifth of the students (22%) Neither agreed nor disagreed with the statement, while less than 10% of the students Somewhat agreed or Strongly agreed.

##### Students agree: the lesson increases their confidence to explain the relationship between enzyme secretion, and sugar transport to cell wall and membrane structure

Question 5: Tell us how much you agree with this statement: After today’s activity, I will be able to explain how enzyme secretion and sugar transport are related to cell wall and membrane function.

We evaluated the students’ confidence in their ability to explain the relationship between enzyme secretion and nutrient transport to cell wall and membrane structure. After the lesson, we asked students to rate how much they disagreed or agreed with the question above. Most students (81.8%) reported that they could explain how enzyme secretion and sugar transport are related to cell wall and membrane function. About 9% of the students were neutral about this statement, while very few of them either “strongly disagreed” (4.5%) or “somewhat disagreed” (4.5%) with the statement (Figure 4B).

### Summative Assessments

#### Readiness Assurance Quiz

During the first 10 min of class, students took a 5-point quiz containing 5 multiple choice questions to test their comprehension of the video lectures (Supplementary Material 1.1). Both cohorts of students performed similarly on the quiz (*t*[176] = 0.44, *p* = 0.66). The Fall 2019 class scored 3.6 ± 1.1 points (mean ± SD, 71%) and the Spring 2020 class scored 3.5 ± 0.9 points (70%). Students were successful at understanding how exoenzymes hydrolyze polymers (questions 3–5) but were less successful in describing where in the cell these processes are happening and how the products of hydrolysis are transported (questions 1–2).

#### Midterm Exam

The midterm questions were designed to assess the students’ mastery of the lesson’s core concepts (Crowe et al., 2008) and have evolved over the development of the course. All exams for this course are open-ended with brief essay responses. For context, after making the course hybrid in Fall 2019, the exams shifted from asking low-order questions to asking students higher-order synthesis questions about the entire process of macromolecule degradation (Supplementary Material 6). In Fall 2018, students averaged 71% on the questions relevant to this lesson, providing basic answers about exoenzyme identification and secretion systems (data not shown). However, students did not demonstrate mastery of the entire macromolecule degradation process. In Spring 2019, students were challenged to describe the degradation process of a protein without the problem-based learning exercise. Their answers averaged 31% for these questions (data not shown). Overall, students from the pre-hybrid course demonstrated a rudimentary understanding of the processes of macromolecule degradation but were challenged when connecting macromolecule degradation to a non-carbohydrate substrate. This demonstrates a breakdown of knowledge with the macromolecule degradation process, thereby inspiring the shift of the questions to the higher-level processes assessed here.

In the exam questions pertaining to this lesson, students are asked to detail the process of decomposition and metabolism of a triglyceride to their final carbon-based products (CO_2_ or a fermentation product). This shift in substrate in the exam, from cellulose used in the lesson, would allow us to ascertain if students transferred the knowledge obtained in the lesson to a new scenario (Nokes-Malach and Mestre, 2013). The exam question had four parts assessing students’ mastery of protein secretion, exoenzyme function, nutrient transport and metabolism. For both semesters, these exam questions were graded by the same grader, using the same key, and the same rubric (Supplementary Material 3). These two cohorts were the first to be assessed with this question.

**FIGURE 5:**
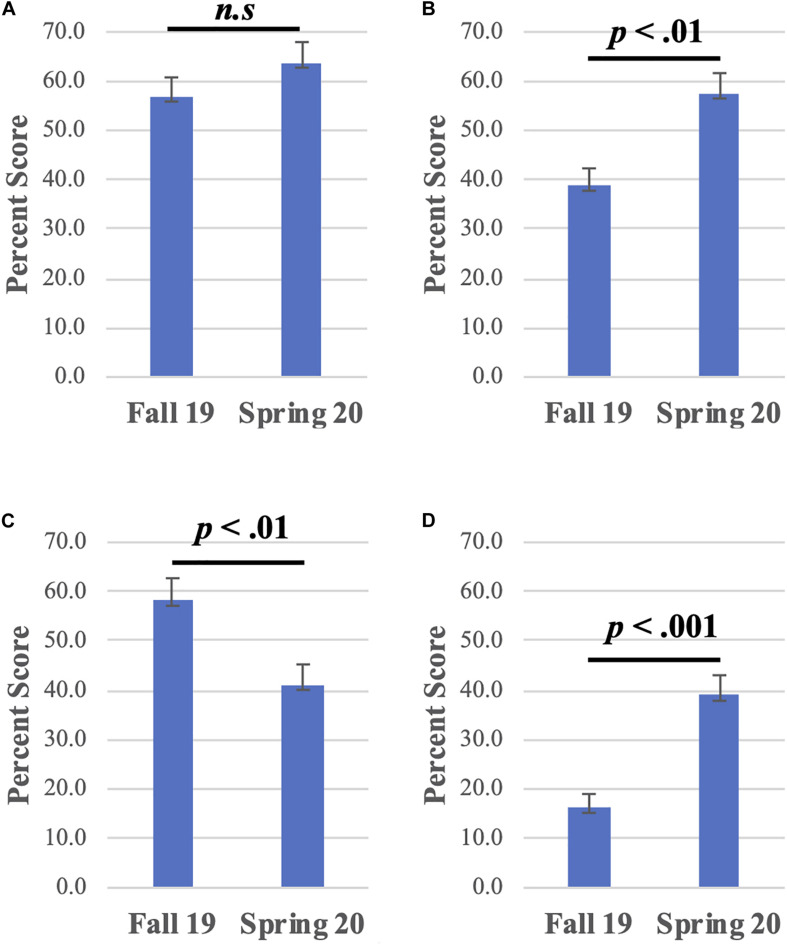
Comparison of exam performance in summative assessment questions for the Fall 2019 and the Spring 2020 cohorts. All graphs compare the mean percentage score and error bars are the standard error of the mean. **(A)** Question 5a: Protein Secretion, *t*(177) = –1.20, *p* = 0.232. **(B)** Question 5b: Exoenzyme Function, *t*(177) = –3.24, *p* = 0.001. **(C)** Question 5c: Nutrient Transport, *t*(177) = 2.63, *p* = 0.009. **(D)** Question 5d, Metabolism, *t*(177) = –4.68, *p* < 0.00001. Bar graphs display the responses for Fall 2019 (*n* = 89) and Spring 2020 semester (*n* = 90). All statistics are unpaired, two-tailed, Student’s *t*-Tests. For details on the questions and their answers, please see the Supplementary Material.

When comparing the entirety of the exam, both cohorts performed similarly (*t*[176] = 1.08, *p* = 0.28). The Fall 2019 cohort had a mean score of 77% and the Spring 2020 had a mean score of 74%. Since Fall 2019, students do not keep their exams after reviewing the material in discussion so there is little chance that the Spring 2020 cohort had access to exam questions ahead of time. Both cohorts demonstrated similar comprehension of the exoenzyme secretion process (Figure 5A). Students in the Spring semester cohort performed better than the Fall semester cohort in the question concerning Exoenzyme function (Figure 5B). On the other hand, Fall semester students performed better in the question relating to nutrient transport (Figure 5C). Although the Spring 2020 students performed better, both cohorts had challenges identifying the products of triglyceride degradation (glycerol and fatty acids) and placing these within the beta oxidation pathway to generate Acetyl-CoA (Figure 5D).

## Discussion

### Practical Implications and Lessons Learned

Peer-led, team-based learning is known to give students an opportunity to develop positive interdependence, scientific reasoning, critical thinking, and communication skills (Scager et al., 2020; Trempy et al., 2002). Additionally, peer-led learning provides a more inclusive and supportive learning environment, particularly to underrepresented students who come from culturally interdependent communities (Covarrubias et al., 2016). General Microbiology utilizes project-based learning in the TEAL classroom environment to facilitate the exploration of real-world scenarios (Dourmashkin et al., 2020) and help students synthesize their own understanding of material (Leupen, 2020). This lesson helped students connect first-hand with the global impact of paper pollution and the role microbes play in the biodegradation of paper waste. Students demonstrated a deep understanding and increased awareness of the societal issues surrounding paper waste management and sustainability.

Illustrating the entire biodegradation process of cellulose was the most challenging part of the activity, as students are required to engage in systems thinking, bringing together the material not only from this lesson, but from two other previous lessons (cell wall structure and nutrient transport). This activity revealed misconceptions in students’ understanding of the material (Figures 2A,C). Some students found it difficult to differentiate between the Sec and Tat protein translocation system, centered around a knowledge breakdown between which system translocated unfolded (Sec) versus folded (Tat) proteins. Furthermore, students were confused by which protein secretion system was used to transport proteins across the outer membrane (Type V Secretion System) and where these secretion systems were located in the cell envelope. This in-class evidence was confirmed through the coding of the post-class metacognitive response to “*What is the most confusing concept in today’s class?*”, where Protein Export was the most commonly represented code, followed by Big Picture and Illustration (Table 2). Another misconception centered on the transport of cellulose, as some students misidentified cellulose as a monomer and missed including glucose in their diagrams. Concerning nutrient metabolism, some students had challenges ascertaining the biochemical pathways utilized to digest glucose monomers as a source of energy. These topics also surfaced in the coding of the answers to the metacognitive question “*What is the most confusing concept in today’s class?*”, where Biochemistry, Nutrient Transport and Exoenzyme Activity codes were abundantly represented (Table 2). Combined, these misconceptions demonstrated a general challenge with visualizing structure/function relationships in the cell envelope. This informed us of the importance of emphasizing the structural components of the lesson for future students. In the next iterations of the class, we will prime students to review these topics during the preparatory stage of the carbon cycle lesson. In this way, the aspects that are least understood to the class would be emphasized during individual lectures and incorporated into activities to solidify this knowledge.

Overall, students were effective at explaining how exoenzymes are secreted (Figure 5A), their role in macromolecule hydrolysis (Figure 5B) as well as the transport of their fatty acid products into the cell (Figure 5C) but were less effective at connecting the biochemical pathway for fatty acid metabolism to energy production. We strategically chose to ask students about triglyceride hydrolysis instead of cellulose to provide them the opportunity to demonstrate their ability to transfer the knowledge they gained from the cellulose lesson to the triglyceride exam question (Nokes-Malach and Mestre, 2013). In this regard, students showed a breakdown of knowledge in the level of completion in their answer regarding the specific pathway by which fatty acids are processed (Figure 5D). For example, some students would correctly state that fatty acids are processed into acetyl-CoA via beta oxidation but could not follow through connecting acetyl-CoA to the Krebs cycle and the electron transport chain. Some students mentioned the Krebs cycle and electron transport chain, but not the beta oxidation pathway. This breakdown may reflect the fact that these questions were the most challenging on the exam and required students to think at a higher-cognitive level than other questions. Moreover, students did not have the opportunity to practice transferring knowledge of the various macromolecule degradation processes from one substrate to another before the exam. This signaled to the instructors to consider providing more opportunities to practice this skill, particularly in the asynchronous and homework. While the exam scores were low, they were an improvement from the scores of the pre-hybrid Spring 2019 cohort, which did not have the aid of the problem-based learning exercise (data not shown). This demonstrates an improvement between the pre-hybrid and hybrid cohorts when approaching higher-cognitive level questions.

Based on their formative assessment results, students showcased evidence of their learning and achievement of the lesson’s learning outcomes. This is illustrated in the transition in predictions about the fate of paper in a landfill (Figure 1C and Supplementary Figure 2). Most students came to class incorrectly believing that respiration played a role in the biodegradation of paper waste products. By the end of the activity, most students stated that fermentation plays a primary role in this process. However, a good proportion of students (41%, Figure 1C) still stated that paper would be degraded via respiratory processes. We hypothesize that the lack of clear understanding of landfill architecture as well as images used during the introduction to the activity of paper exposed to air in landfills might have influenced student’s answers (Supplementary Material 1.2). In future iterations of the class, we will ensure to reinforce these concepts in the pre-flipped lecture preparation material.

Students were able to visually work through the mechanism of nutrient transport and correctly illustrate the biochemical processes accurately. Misconceptions of the structural organization of these processes were identified quickly and resolved through visual exploration and discussion (Schnotz, 2014). Our metacognition survey demonstrated that students self-identified their growth in understanding before and after the activity (Figure 4B). The students synthesized and demonstrated their understanding through the summative assessments, being able to recall the process of protein secretion and nutrient absorption but struggled to transfer knowledge from one polymer (cellulose) to another (triglycerides). Future class iterations will provide discussion forum questions and homework that will give students more opportunities to examine a variety of macromolecules and work through the entire biochemical pathways needed for their degradation.

### Conclusions and Recommendations

Using a variety of teaching strategies and technology, this lesson departed from a traditional teaching model by giving students an opportunity to address a real-world problem. Students demonstrated their learning by building their own systems-level understanding of the microbiological and biochemical processes involved in the breakdown of paper. This lesson was facilitated by, and took advantage of, the TEAL lab learning environment and a hybrid online model, both components that may not be accessible to all universities. The TEAL lab facilitates the use of active learning, but it is neither required to use these strategies nor required to create a collaborate learning environment. There are many ways to foster collaborative learning including structured discussion, reciprocal teaching, and problem-solving (Barkley et al., 2014) that are independent of the TEAL lab. For courses that do not have access to online resources, face-to-face interactions can produce statistically similar grades from online learning as well as increased levels of student satisfaction (Summers et al., 2005).

There are some inherent challenges that come with implementing a flipped lesson with evidence-based teaching strategies of this nature. It takes a significant amount of time to prepare each component, which requires intention and alignment with the learning outcomes to be effective for student learning and engagement. Additionally, these active learning strategies may be new to instructors accustomed to traditional teaching and may require additional training to model effectively. To facilitate the amount of activity in the given time, the timing during flipped lecture needs to be managed closely to incorporate all the components of a 5E model lesson. Timing itself could be a limitation if courses are less than the 75 min discussed here and if instructors feel there is not enough time to cover the content with active learning (Graffam, 2007). This lesson may be difficult to implement in a large (>200 person) class and may require additional levels of organization or facilitation. Furthermore, there may be systemic resistance to incorporating active learning techniques (Bathgate et al., 2019) that faculty may need to overcome without reward (Michael, 2007).

In order to increase content retention and reduce misconceptions, students could perform a pre-lecture homework where they can review the materials discussed in the lesson’s video lectures as well as from previous coursework. We found that, overall, students improved their understanding of the concepts after the 5E lesson. Where some students struggled was drawing the structures related to the biological processes. We recommend that instructors incorporate more model drawing activities to help identify misconceptions and provide students with an opportunity to promote reasoning skills (Quillin and Thomas, 2015). We also recommend offering a variety of examples for students to practice transferring their knowledge from one example to another. Lastly, we recommend and encourage faculty to center activities, when possible, on real-world scenarios that are relevant to students, especially scenarios that impact underserved student communities, to connect the concepts and their implications to student experiences (Harackiewicz et al., 2016).

## Summary

Today’s students suffer from the burden of climate change and global pollution and need to develop skills to think critically about these problems. Problem-based learning can engage students in real-world scenarios while simultaneously learning complex microbiological and biochemical concepts. By using deliberate teaching strategies, outlined in this lesson, we demonstrate increased student conceptual understanding and perceived understanding of microbial carbon assimilation and its role in paper waste degradation. We coded student responses from a reflective survey and identified common misconceptions and perceived gains. Student performance was measured through a variety of assessments including a drawing activity and exam questions. We were able to determine areas where student performance could improve and address them accordingly. We recommend instructors consider using real-world scenarios when teaching complex topics to foster student engagement and interest in the topic in and beyond the classroom.

## Data Availability Statement

The raw data supporting the conclusions of this article will be made available by the authors, without undue reservation, to any qualified researcher.

## Ethics Statement

The studies involving human participants were reviewed and approved by UC Merced Institutional Review Board. The patients/participants provided their written informed consent to participate in this study. Written informed consent was obtained from the individual(s) for the publication of any potentially identifiable images or data included in this article.

## Author Contributions

MG-O conceived the lesson’s idea with assistance from JS. MG-O and JS developed the learning outcomes, hybrid flipped classroom format, and created course materials such as the video lectures and assessments. MG-O and RS developed the codebook and coded the metacognitive responses. MG-O created and edited figures and tables as well as performed the statistical analyses. JS wrote the manuscript with contributions from MG-O and RS with editing and revisions from MG-O. All authors discussed the results and contributed to development of the manuscript.

## Conflict of Interest

The authors declare that the research was conducted in the absence of any commercial or financial relationships that could be construed as a potential conflict of interest.

## Footnotes

1 http://wordclouds.com/

## Abbreviations

ERI: emergency remote instruction
LMS: learning management system
PTS: phosphotransferase system
sec system: secretion system
STEM: science technology engineering and mathematics
tat system: twin-arginine translocation system
TEAL: technology enabled active learning.
