# Supplementary material for: Tackling Real-World Environmental Paper Pollution: A Problem-Based Microbiology Lesson About Carbon Assimilation
Source: Front Microbiol. 2020 Nov 5;11:588918. doi: 10.3389/fmicb.2020.588918 (PMC7674769; doi:10.3389/fmicb.2020.588918)
Supplement: Supplementary file 1 [file Data_Sheet_1.PDF]

Supplementary Material

1 Supplementary Material

Supplementary Material 1. Course content for General Microbiology (BIO120)

We use the Brock Biology of Microorganisms (Madigan et al. 2018, 15th edition). The carbon assimilation content comes from Ch. 3 (sections 3.3–3.7), Ch. 4 (sections 4.11–4.13), Ch. 14 (sections 14.24–14.25), Ch. 21 (sections 21.1& 21.8), and Ch. 22 (sections 22.4–22.5).

Supplementary Material 1.1 Carbon Acquisition-Video Lecture Slides

Supplementary Material 1.2 Session 12 Leaning Outcomes-Flipped Lecture Slides

Supplementary Material 2. Lecture Comprehension Quiz

1. The Sec secretion system is able to transport proteins into the periplasmic space. Which of the following statements is true?
    a. It translocates folded proteins to the periplasmic space.
    b. **It requires the Type V Secretion System to secrete proteins to the extracellular space.**
    c. Proteins are translocated while they are being translated.
    d. This system uses the proton motive force to secrete proteins.
    e. The sec system is not involved in placing proteins in the membrane.
2. Exoenzymes hydrolyze polymers at the exo-position.
    a. True
    b. **False**
3. Which of the following is true?
    a. **Hydrocarbons are hydrolyzed into acetyl-CoA by beta-oxidation.**
    b. Hydrocarbons can be catabolized directly, without modification, by hydrolytic enzymes.
    c. Dioxigenases are needed to modify hydrocarbons.
    d. Hydrocarbons cannot be use by bacteria to harvest energy.
    e. Ring activation reactions are needed to hydrolyze the hydrocarbon octane.
4. Match the following enzymatic reactions to their respective enzymes. (Word bank: lipase, nuclease, proteinase, amylase)
    a. Hydrolysis of proteins
    b. Hydrolysis of DNA
    c. Hydrolysis of phospholipids
    d. Hydrolysis of starch
5. What is the function of "converter" enzymes?
    a. **They process precursor molecules so the products can then enter central metabolic pathways.**
    b. They catalyze reaction for energy extraction.
    c. They convert amino acids into carbohydrates.
    d. They are involved in transport of nutrients.

Supplementary Material 3. Midterm Questions

5. Mono Lake harbors millions of Alkali flies *Ephydra hians*. The larvae of these flies are rich in triglycerides (three fatty acids bound to a glycerol molecule, see image). Microorganisms decompose dead alkali fly pupae and gather energy and carbon from triglycerides. (*24 points*)

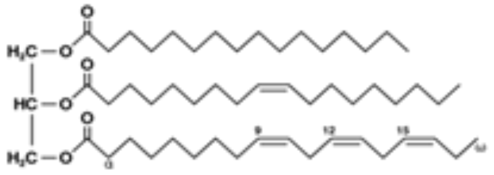

a. Briefly describe where in the cell these exoenzymes are made and the process by which these exoenzymes are secreted. **(Protein Secretion Question)**

Key: Exoenzymes are made in the cytoplasm and then transported to the exterior of the cell via a Secretion System (SS). Here students might mention the Sec-dependent Type II or type V SS, which allow the exoenzymes to be released from the periplasm onto the extracellular milieu. Alternatively, they might mention the Sec-independent systems: Type I, III, IV.

This question is worth 8 points. Students receive 2 points for identifying that the cytoplasm is where exoenzymes are made. They receive 2 points for mentioning the secretion system, and up to 4 points for describing the secretion system details.

b. Name the exoenzyme involved and describe the exoenzyme-mediated events that occur outside the bacterium. **(Exoenzyme Function Question)**

Key: Lipases will break down triglycerides into fatty acids and glycerol.

This question is worth 2 points. Students receive 1 point for correctly identifying the enzyme lipases and the other point is given for identifying that lipases are broken down into fatty acid and glycerol.

c. How are the products of these hydrolytic reactions transported into the cytoplasm of the bacterium (for this question, ignore the glycerol)? Do not forget to mention the energy used for transport. **(Nutrient Transport Question)**

Key: Fatty acids will be transported inside the bacteria by ABC transporters. Binding proteins bind to the FA which are then transported by the ABC transporter after ATP hydrolysis.

This question is worth 4 points. Students receive 2 points for identifying the ABC transporter system as the method of transport and receive the additional 2 points for correctly describing how this system works.

d. Once inside the cytoplasm, the products of triglyceride hydrolysis are used to generate energy. In general terms, describe the process used to harness energy from the products of triglyceride hydrolysis, all the products generated, and the biochemical fate of these products (i.e. to which biochemical pathway these products go). **(Metabolism Question)**

Key: Fatty acids are hydrolyzed via beta-oxidation. The products of beta oxidation are Acetyl-CoA, NADH and FADH2. The Acetyl-CoA will go onto the Krebs cycle while the NADH/FADH2 will go to the ETC.

This question is worth 10 points. Students receive the maximum points for outlining the entire process from beginning to end. If students are only missing one process, they receive 8 points. If they only describe two of the processes, they receive 6 points. If students only describe one process, in detail, they receive 4 points. Most students are able to at least describe some part of this process, receiving between 4–10 points.

Supplementary Material 4. Metacognition code book

Category	Codes	Description	Sample
Concepts	Biochemistry	Student expresses confusion about a biochemical pathway, including (but not limited to) hydrolysis of glucose via glycolysis (or another glycolytic pathway), fermentation, or respiration.	The most confusing concept today was the biochemical process that lead to metabolism of these products and how they go in.
	Exoenzyme Activities	Student had challenges understanding how exoenzymes break down macromolecules, or identifying which exoenzyme is required to hydrolyze a macromolecule.	Today's most confusing topic involved understanding the role of exoenzymes. Particularly the role of exoenzymes during the degradation of cardboard.
	Location	Student expresses confusion about the cellular location of a biomolecule (DNA, RNA, lipid, carbohydrate or protein). This includes not placing a biomolecule in its correct subcellular location.	When assigning the group the drawing activity I was overthinking the assignment. I am little lost on the placement of the protein complex at the cell membrane.
	Misconception	The student expresses a statement that is incorrect as a result of faulty thinking or lack of understanding the activity. The student mixes up concepts that are not usually related or confuse the order of biological or biochemical events.	**I had a hard time understanding how exoenzymes can secrete paper.** I looked it up for various minutes and most of the scholarly articles stated that exoenzymes did secrete paper but not exactly how.
			I didn't understand what was being asked when we were asked to draw the transport mechanisms in the bacterial membrane. I think that's when I was the most confused in class.
	Nutrient Transport	Students had challenges determining how a nutrient (glucose) would be transported from the outside to the inside of the cell. This includes crossing the outer membrane of the gram negative cell wall (via porins) as well as the cell membrane (via PTS system, ABC transproters or permeases).	The transportation of the products of exoenzyme reactions was confusing to me because it had a lot of stuff going on with it, including the different transporters and the conditions (like binding and phosphorylation and such)
	Protein Export	Students had challenges determining how proteins (exoenzymes) would be transported from the inside of cell to the outside of the cell. This includes distinguishing which protein system are used to translocate proteins across the cell membrane (via Sec or Tat systems) or used to cross the gram negative cell wall (via protein secretion systems).	The most confusing concept was understanding how exoenzymes are secreted to the environment.
	Other	Any type of statement that does not fit in the previously described categories. This includes non-content statements.	The most confusing topic today is understanding the agent and environment interventions.
Competencies	Big Picture	Student expresses challenges connecting key concepts from various lectures, or demonstrating understanding of the cell as a system. This would include linking the topic to the various spheres of biological influence.	The most confusing concept was trying to link the past 3 lectures into the activity where we drew how cellulose broke down and was taken into the cell
	Illustration	Students had challenges illustrating, drawing and organizing a model based on their mental vizualization of the concepts.	The most confusing concept today was being able to illustrate the process by drawing and labeling what is happening outside and inside of the membrane.
	Time	Students states that time constraints were a factor in their lack of understanding of the activity.	When doing the last activity it felt rushed and I wish we could've gone through it together as a class.
Affect	Concern	Student expresses concern about their confusion or lack of knowledge, or how it would affect their performance in an assessment.	Understanding and being able to connect previous lectures into answering possible exam questions about biodegradation. **I understood the paper waste degradations from my peers but I am still worried if I am able to recount what I learned in future exam questions.**
	Improvement	Student expresseses a positive view about their learning or about seeking help from a peer, study group, tutor, TA or professor. This includes the **desire** to gain better understanding of a topic or proposing an autonomous or collective (group work) way to gain mastery of the material.	Drawing the diagram was the most confusing concept today. **Understanding how the various parts work together was tricky, but I know if I keep reviewing it outside of class and watch videos on the mechanism, I will be able to fully understand it.**

Supplementary Material 5. Asynchronous online discussion forum questions

Students participated in an asynchronous online discussion during the same week they watched their videos and performed their carbon degradation activity. Discussion question are posted for a week where students have time to choose which questions they like to answer and write a post of at least 250 words with references. They must also reply to a peer, offering further questions or clarifications on ideas. The discussion is facilitated by co-instructor (Shay) to moderate for any misconceptions or misdirected content. The discussion questions related to this activity are as follows:

- **Transporters**: Choose two different transport methods (e.g. passive diffusion, PTS, ABC transporter, or other examples from the lecture) and compare and contrast the two. Make sure to comment on the purpose of this method of transport, any parts involved, enzymes, the energy required, and how it works.
- **Exoenzymes**: What are exoenzymes? How do they work? Why do microbes have them and what purpose do they serve? Provide specific examples from class materials to support your ideas.
- **Degradation**: Microbes have incredible abilities to degrade toxic substances like petroleum and oil. How do they manage to do this? What processes are involved? What are the implications for these processes and how could we attempt to harness this power to save our future? Think critically about this prompt using your course materials.
- **Fermentation**: What is the overall purpose of fermentation? Explain the differences and similarities between two types of fermentation of your choosing. Make sure to note the inputs and outputs of the reaction and the energy consumed/used or gained in the reaction.

Supplementary Material 6. Pre-Hybrid exam questions and key

<u>Fall 2018 Exam Questions</u>

5. The cyanobacteria *Geitlerinema terebriformis* live in biofilms made of complex cellulose-like polymers. These can provide energy and carbon sources when digested by exoenzymes.

a. Name the type of exoenzymes used to digest these polymers. Briefly describe the process by which these exoenzymes are secreted (10 pts).

> **Key: Cellulases will be secreted by the bacteria onto its environment. These proteins will be transported from the cytoplasm to the periplasm during biosynthesis through the Sec system. Type II, IV, and V secretion systems will allow the cellulases to be released from the periplasm onto the extracellular milieu.**

b. Describe the exoenzyme-mediated events that occur outside the bacterium as well as how the products of these events are transported into the cytoplasm of the bacterium (8 pts).

> **Key: Cellulases will break down the polymer into glucose monomers. These will then be transported inside the bacteria by a PTS transport system**.

<u>Spring 2019 Exam Questions</u>

5. Following protein degradation, *V. cholerae* can import the amino acids Alanine and Glycine into its cytoplasm for energy production.

a. **Discuss** the biochemical modification of Alanine to form a **3-carbon product** that will be used eventually in aerobic respiration. In your answer name the family of **enzymes** that catalyze this modification, and the **byproducts** of this reactions (6 pts).

Key: Deamination of Alanine (1) into pyruvate and NH3 (2) via deaminases converter enzymes (2)

b. **Discuss** the biochemical co-modification of Alanine and Glycine to form a **2-carbon waste product** that will be secreted eventually under anaerobic conditions. What is the Nitrogen containing product also made in these reactions? (6 pts)

Key: Strickland reactions with oxidation of Alanine (1) coupled with reduction of glycine (1) via NAD+ shuttling of electrons (2) results in pyruvate and NH3 (2)

2 Supplementary Figures and Tables

2.1 Supplementary Figures

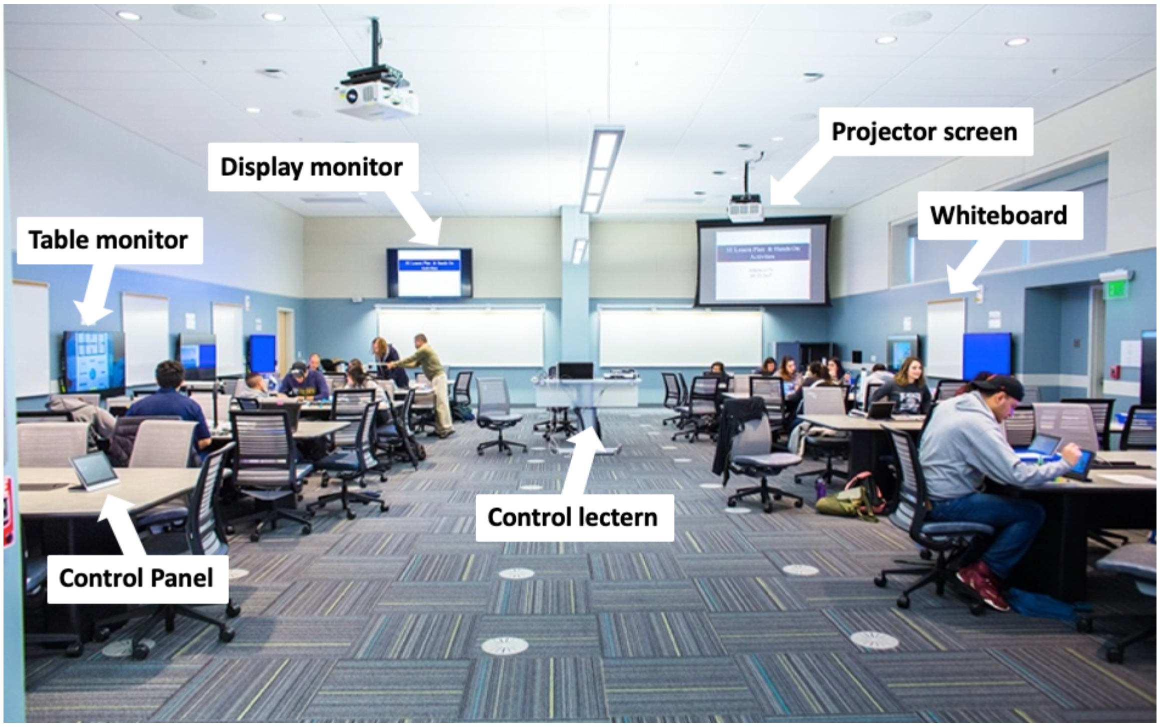

Supplementary Figure 1. UC Merced Technology-Enabled Active Learning (TEAL) Lab. This learning space facilitates collaborative discussions, simulations, and hands-on learning experiences. The image illustrates a 90-seat lab divided in 10 tables (9 students per table). Each table has a LED HDMI screen and a white board as well as connectivity for laptops and document cameras. Front and back projectors and screens ensure that all students have direct visual access to class material. Instructors control the room via a central lectern.

A)

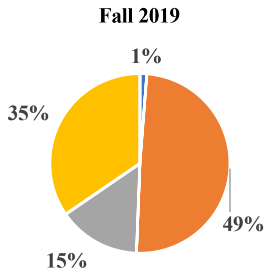

B)

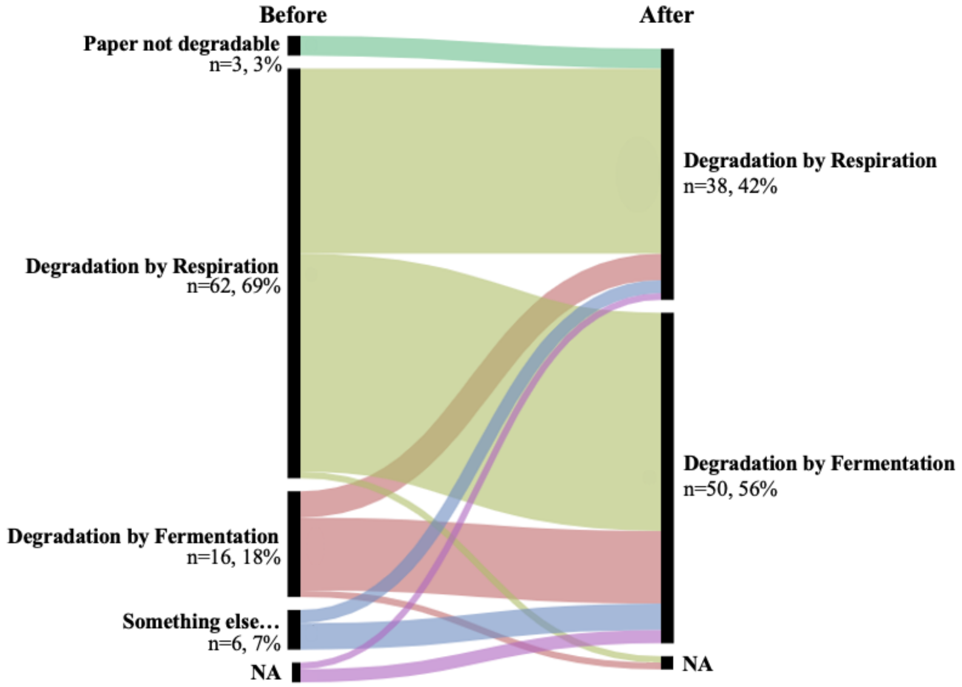

Supplementary Figure 2. Students' prediction about the fate of paper in a landfill. A) After a brief introduction, Fall 2019 students (n = 81) were asked to predict the fate of paper in a landfill. This 5-point Likert survey question was deployed via clickers before the activity. The answer choices included "*It remains in the landfill, as paper is not degradable.*" (blue), "*It will decompose by microbial activity involving respiration.*" (orange), "*It will decompose by microbial activity involving fermentation.*" (gray), and "*Something else will happen...*" (yellow).

B) Comparison of student's predictions before and after the activity for Spring 2020. Before and after the activity, Spring 2020 students (n = 88) were asked to predict the fate of paper in a landfill. This 5-point Likert survey question was deployed via clickers before the activity or via the Learning Management System after the class. The answer choices included "*It remains in the landfill, as paper is not degradable,*" "*It will decompose by microbial activity involving respiration,*" "*It will decompose by microbial activity involving fermentation,*" and "*Something else will happen...*" The alluvial plot compares the before (left column) and after (right column) responses.

2.2 Supplementary Tables

Supplemental Table 1. Lesson timeframe.

Lesson Component	Time[1]	Slide[2]	Notes:
Welcome to Class	**-10**	N/A	The classroom is available 10 minutes before class starts. This time allows welcoming the students to the learning environment.
Readiness Quiz	8	N/A	This quiz can be taking online prior to attending the flipped lecture. After the class was transitioned online due to the Covid-19 pandemic, this was the preferred method to administer quizzes.
Lesson's learning outcomes	2	1	Quick reminder of the lesson's learning outcomes
Introduction 1: recycling	5	2	Introduction to the National Sword Policy and its impact in paper recycling.
Recycling Discussion and questions	5	2	Student's answer the question "What type of paper products would be rejected by the National Sword policy?"
Introduction 2: paper origins and paper waste management	5	3	Introduction to the nature of paper production and general overview of paper waste management.
Clicker question 1	3	4	We provide approximately 1 minute to students to think about the question. The clicker question data collection takes 1 minute. The results of this question are not discussed but we state that we are collecting this information to compare their responses at the end of the lesson.
Clicker prediction 1	3	5	I provide approximately 1 minute to students to think about the question and 1 minute to answer the question. The results are not discussed.
Group Exercise 1: Research	10	6	Students research and answer question about the molecular composition of paper, microorganisms that degrade cellulose, the exoenzymes used, exoenzyme secretion, nutrient transport and metabolic reactions. This is done as a jigsaw puzzle, where the group is divided in 2 research pods.
Class discussion on Group Exercise 1	10	6	This time allows students to review and share their answers to the questions in slide 6. This allows us time to identify misconceptions that could have come up during their research.
Group exercise 2: Illustration	15	7	Students are verbally instructed to illustrate the process of exoenzyme secretion, cellulose hydrolysis, glucose transport across the cell envelope and glycolysis. The instructors and learning assistant monitor the progress of all groups and provide feedback when misconceptions are identified.
Clicker prediction 2	2	8	I provide approximately 1 minute to students to think about the question. The clicker question data collection takes 1 minute. The results of this question are not discussed but we state, that we are collecting this information to compare their responses at the end of the lesson.
Class Closure	2		Students conclude the class and are reminded about the online metacognitive surveys.
Outside of class	**-10**		Student answer survey with metacognitive questions. The question in Clicker Prediction 2 can be incorporated in this survey to save time.
Total in class time	70		This time does not include the Outside class nor the Welcome to class time.

[1]Time in minutes. [2]Slide numbers of Supplemental Materials 1.2.

Supplemental Table 2. Word cloud term occurrence data.

Based on today's work, tell us what you think about the following statement: The power of microbes can be harnessed to reach environmentally sustainable goals.		Does today's work illustrate the relationship of microbiology to society? Explain.	
**Term**	**Weight**	**Term**	**Weight**
**microbes**	**74**	microbes	72
**environment**	**35**	waste	39
waste	30	**society**	**38**
help	26	help	32
degradation	24	paper	27
**sustainable**	**24**	degradation	26
break	12	environment	25
**goals**	**11**	landfill	23
paper	11	recycling	23
landfill	10	**microbiology**	**20**
recycle	10	trash	19
clean	8	break	16
decompose	8	important	14
microorganisms	8	pollution	13
bioremediation	7	problems	13
oil spills	7	microorganisms	12
plastic	7	decomposition	10
produce energy	7	sustainable	10
cardboard	6	relationship	9
pollution	6	cardboard	7
soil	6	increase	7
solution	6	life	7
world	6	products	7
carbon	5	humans	6
natural	5	knowledge	6
nitrogen	5	learn	6
biodegradation	4	people	6
exoenzymes	4	population	6
materials	4	production	6
people	4	solution	6
