# Supplementary material for: Tackling Real-World Environmental Paper Pollution: A Problem-Based Microbiology Lesson About Carbon Assimilation
Source: Front Microbiol. 2020 Nov 5;11:588918. doi: 10.3389/fmicb.2020.588918 (PMC7674769; doi:10.3389/fmicb.2020.588918)
Supplement: Supplementary file 2 [file Data_Sheet_2.PDF]

Carbon Acquisition

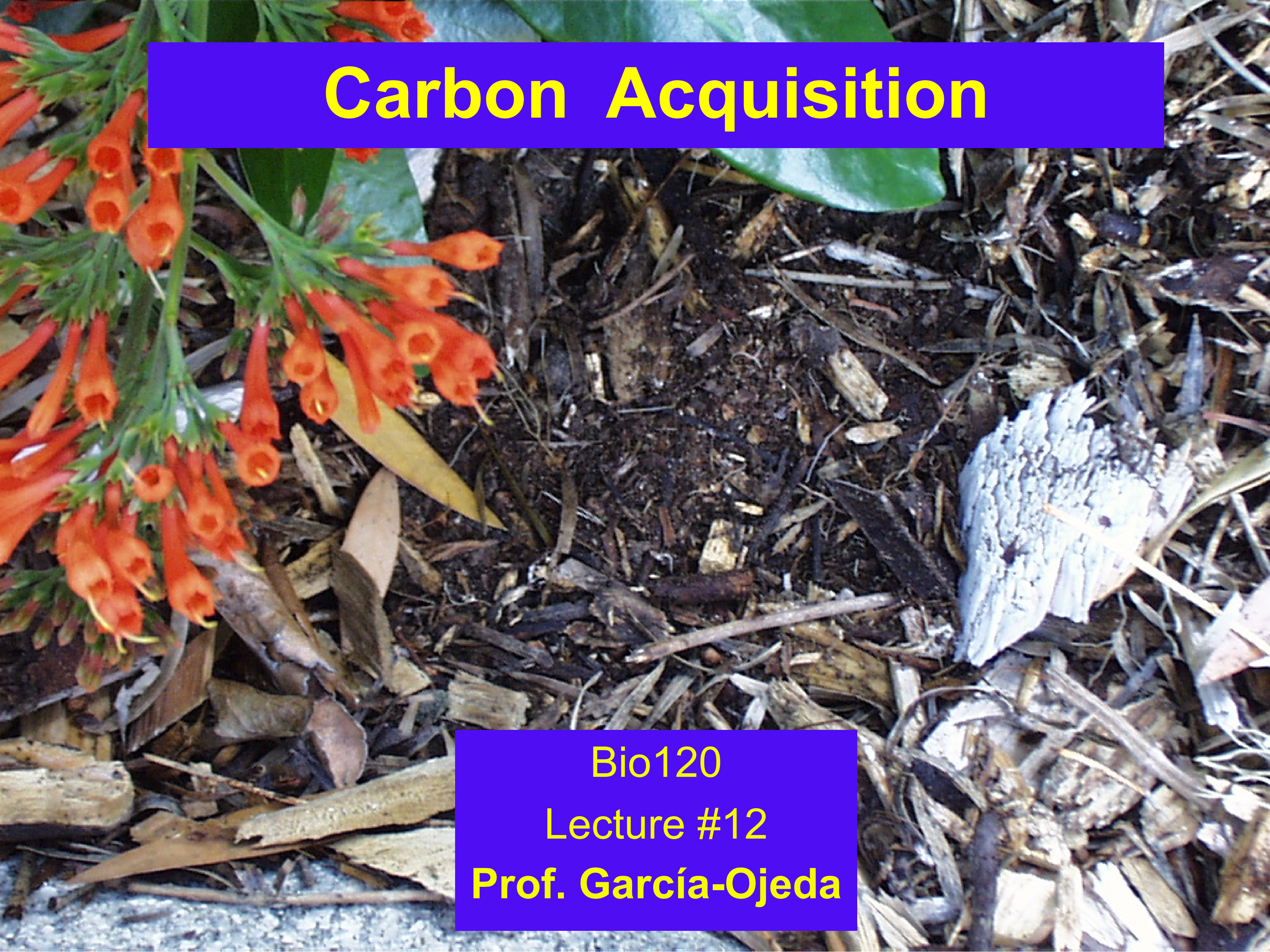

Bio120

Lecture #12

Prof. García-Ojeda

Bringing carbon into biological molecules.

THE CARBON CYCLE

Organic Carbon on Earth Cycles

Microbes play a critical role in carbon fixation:
- by photosynthesis
- by chemosynthesis

Microbes recycle organic carbon by decomposition

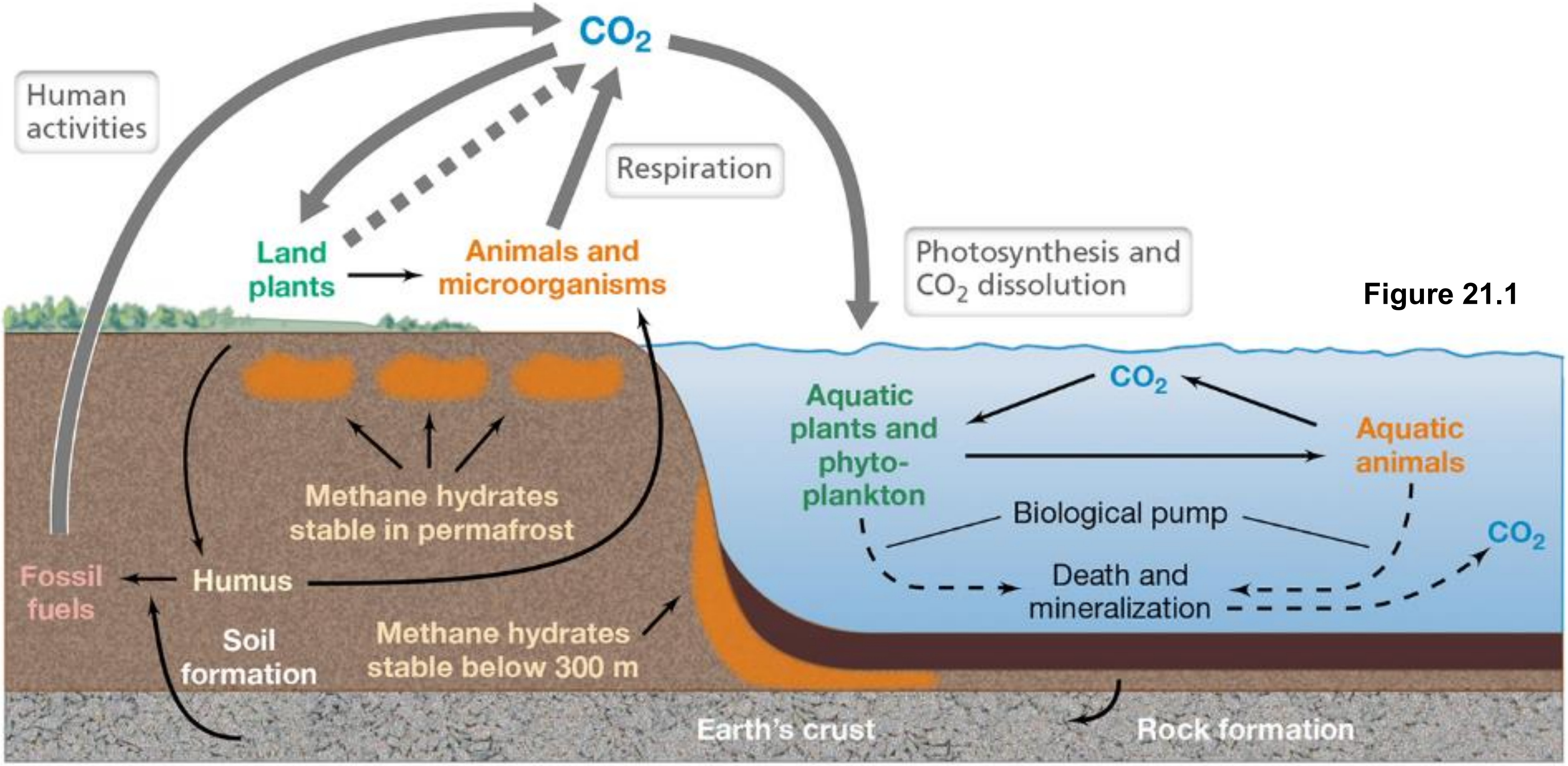

Figure 21.1

Most carbon is inorganic in the form of rocks & sediment (99.5%) .

The rest is CO_2 or organic carbon derived from living cells (0.5%).

The organic carbon on Earth is present as:

- small bio-molecules

substrates, intermediates & cell waste

- complex biomolecules

polymers such as protein, DNA, carbohydrate

- geological carbon

coal, gas, oil & methane hydrates

- man-made compounds

plastics, pesticides, herbicides, drugs, and other synthetic compounds

Major Carbon Reservoirs on Earth

Reservoir	Percent of Total[a]
Rocks and sediments	99.5[b]
Oceans	0.05
Methane hydrates	0.014
Fossil fuels	0.006
Terrestrial biosphere	0.003
Aquatic biosphere	0.000002

[a]Total carbon, 76×10^{15} tons
[b]80% inorganic

Fig 21.1

Fate of Organic Carbon on Earth

An excellent generalization…

For <u>every</u> **naturally** occurring organic compound found in nature,….

…some microbe has evolved a way to degrade it!

…as a source of cell energy, and/or

….as a source of carbon.

(it is recycled back to CO_2)

Organic compounds are generated by photosynthesis and broken down by respiration

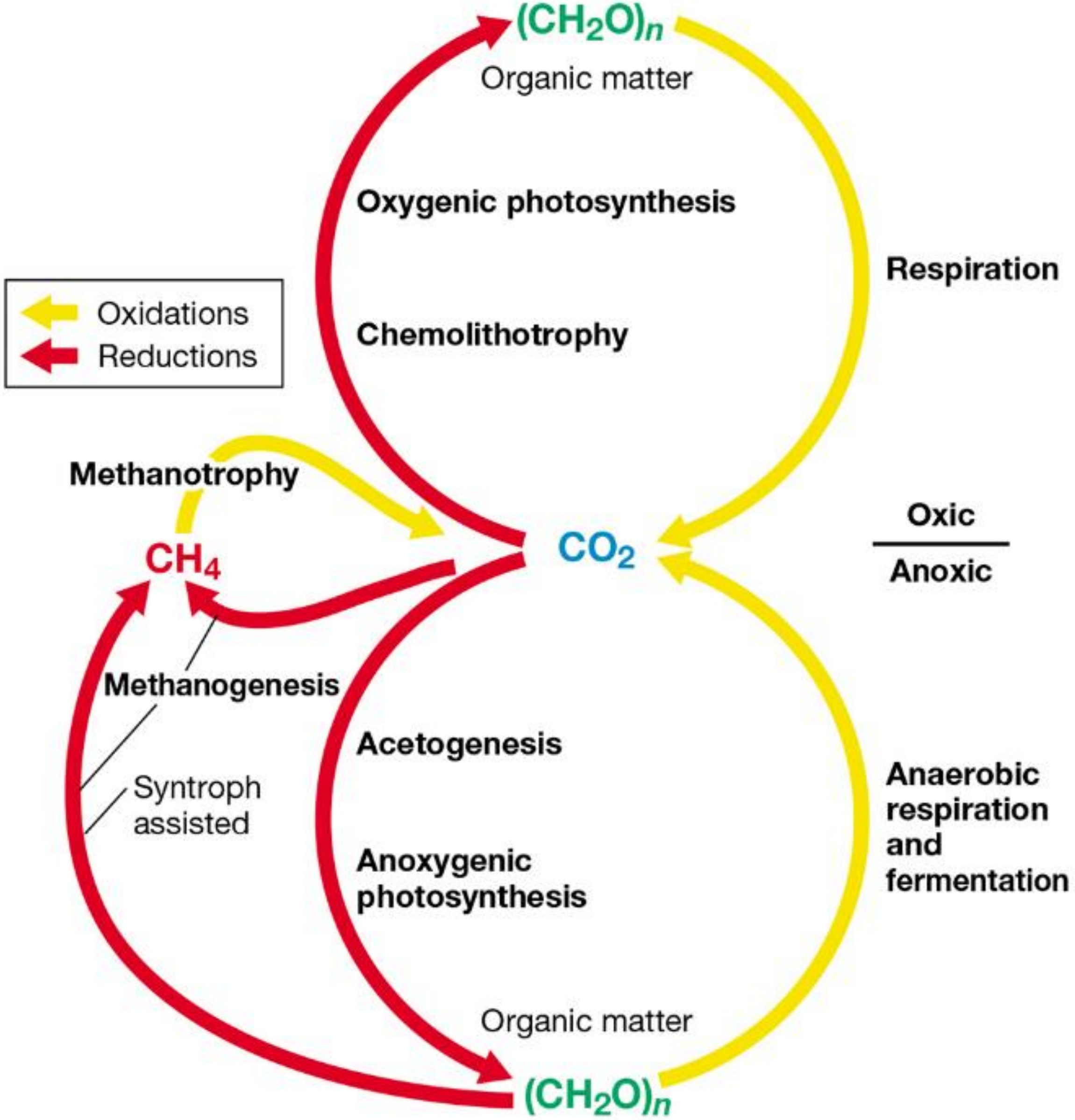

Figure 21.2

All nutrient cycles are “coupled”, where changes in one affect another

- Primary production refers to the rate of CO_2 fixation during photosynthesis
- The rate of primary production is controlled by the magnitude of photosynthetic biomass and the availability of N.

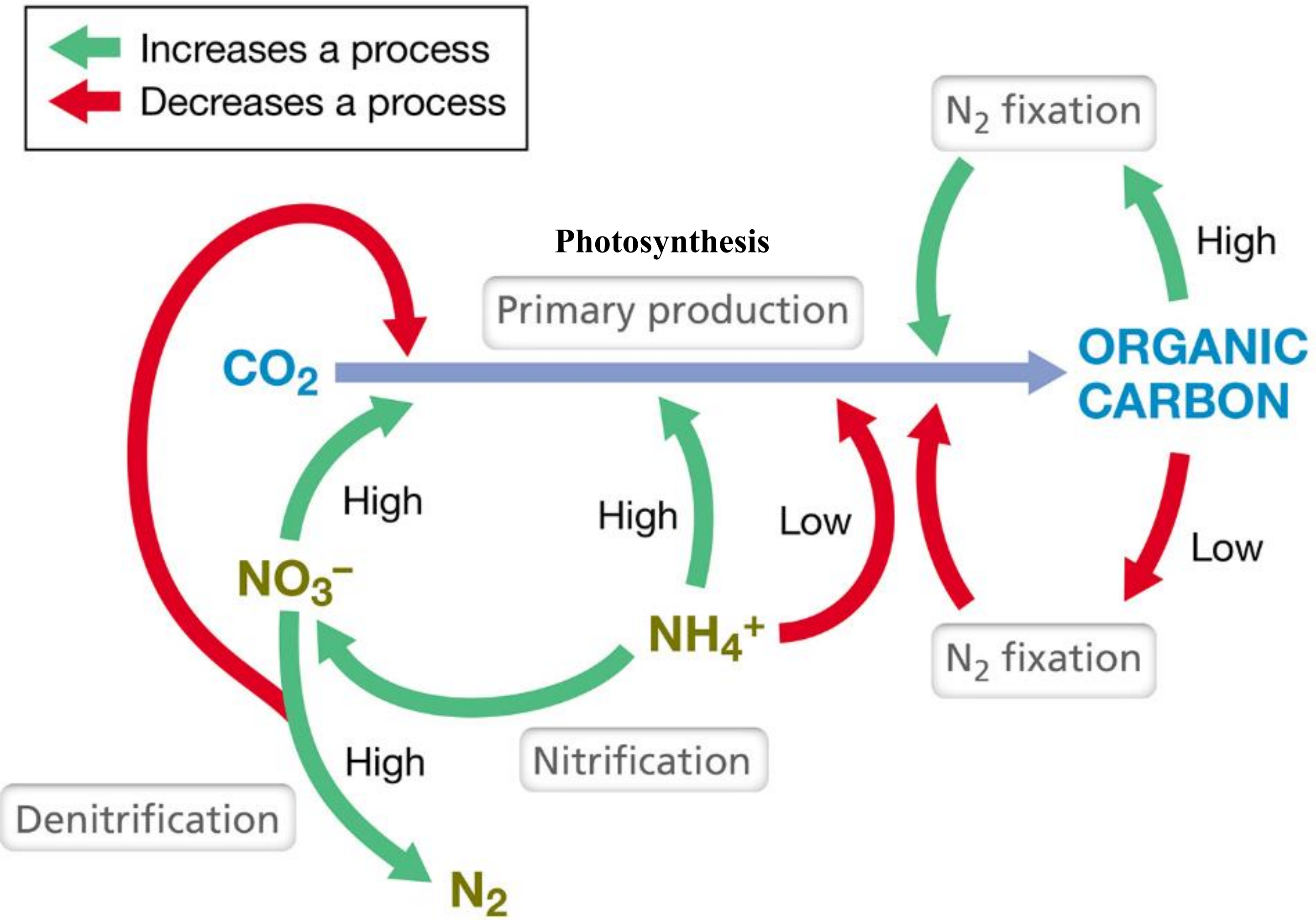

Fig. 21.5

Organic material in soil particles

dead, dying, and decaying plant and animal material, animal droppings, insects, worms, microorganisms.

Most of the biomass is **polymeric!**

polymers of:

- complex polysaccharides
- proteins
- nucleic acids
- lipids
- lignin

    complex compounds derived from wood and an integral part of the plant cell wall.

- oils and waxes

(small molecules are only about 5% of total cell mass!)

However, microorganisms cannot chew, eat, absorb, or engulf

- other living or dead organisms,
- small particles of food,
- macromolecules.

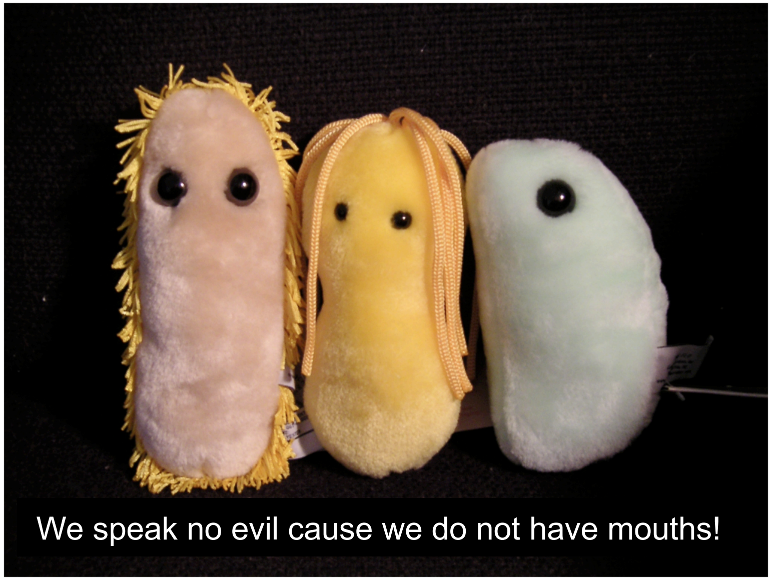

Therefore, polymers must be degraded outside the cell by exo-enzymes.

Exo-enzymes are secreted!

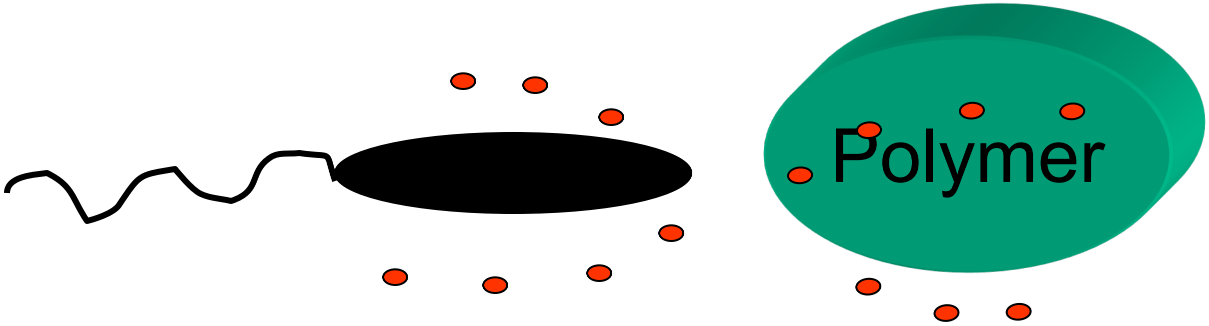

Exo-enzymes attack the polymers, breaking their bonds by hydrolysis.

for example:

cellulase attacks cellulose at β-1,4 linkages, hydrolyzing this bond.

- The resulting products are transported into the cell!

Next: Protein Secretion

END OF CARBON CYCLE

How exo-enzymes and other proteins are **exported** by microbes.

PROTEIN SECRETION

Sec-dependent protein secretion

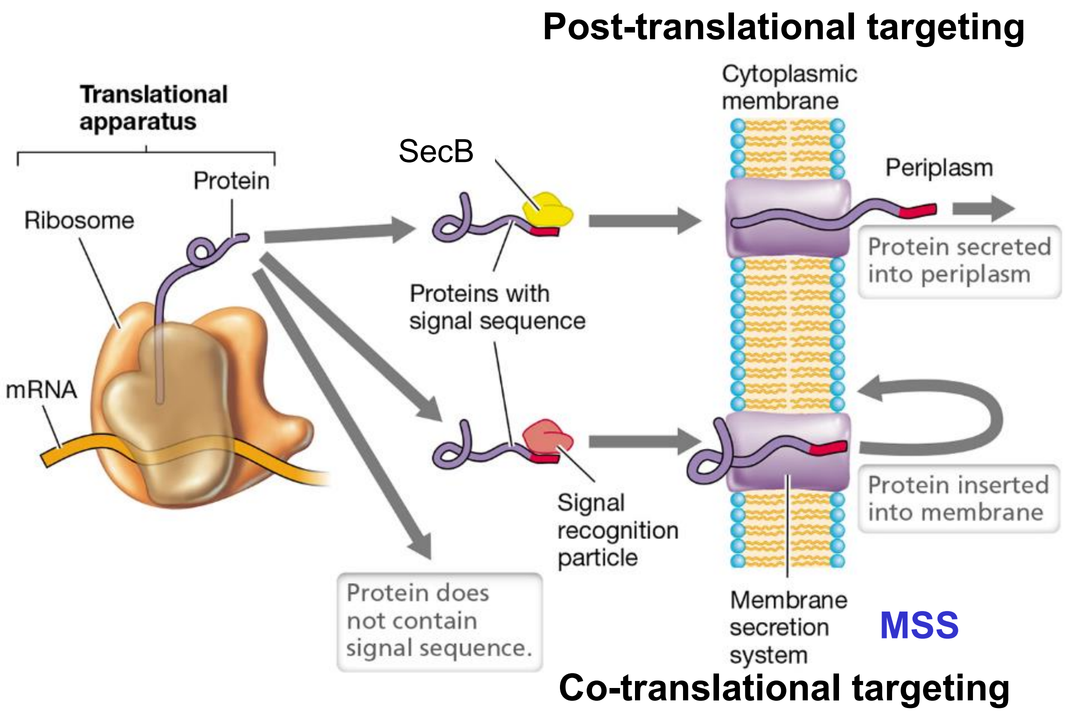

- SecB brings the protein to the SecA MSS.
- Sec-dependent system requires ATP hydrolysis by SecA for translocation.
- Once translocated, the enzyme **signal peptidase** removes the signal peptide.
  - The protein then folds into its proper shape.

- Found in **all** domains of life
  - Translocates **unfolded** proteins across the plasma membrane
  - it is responsible for the secretion of most extracellular proteins
- Proteins are synthesized as pre-proteins with a signal peptide
- During synthesis, SecB works as a chaperone, binding to the nascent signal peptide and stabilizing the protein.

Tat- and SRP-dependent protein secretion

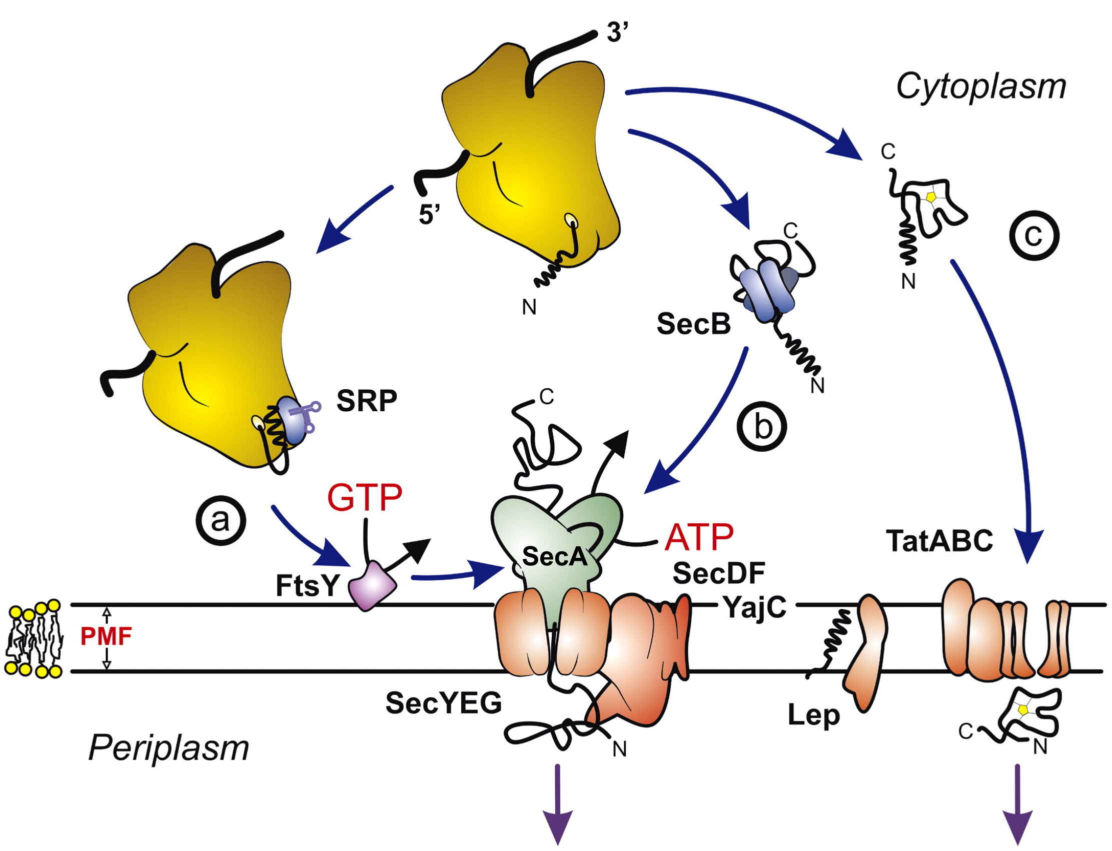

Biochimica et Biophysica Acta – Biomembranes (2008). 1778 (9):1735-1756

- Tat for "twin-arginine translocase"
  - The Arg-Arg motif is found on the signal sequence recognized by the Tat-translocase.
- Transports small proteins that **fold** prior to translocation.
  - Usually proteins bound by co-factors, such as cytochormes
- Uses the proton motive force for translocation.

- SRP (signal recognition particle) binds to proteins destined to be **embedded** in the membrane.
  - Similar to the eukaryotic **co-translational** secretion system.
  - Uses Sec-channel to translocate proteins.

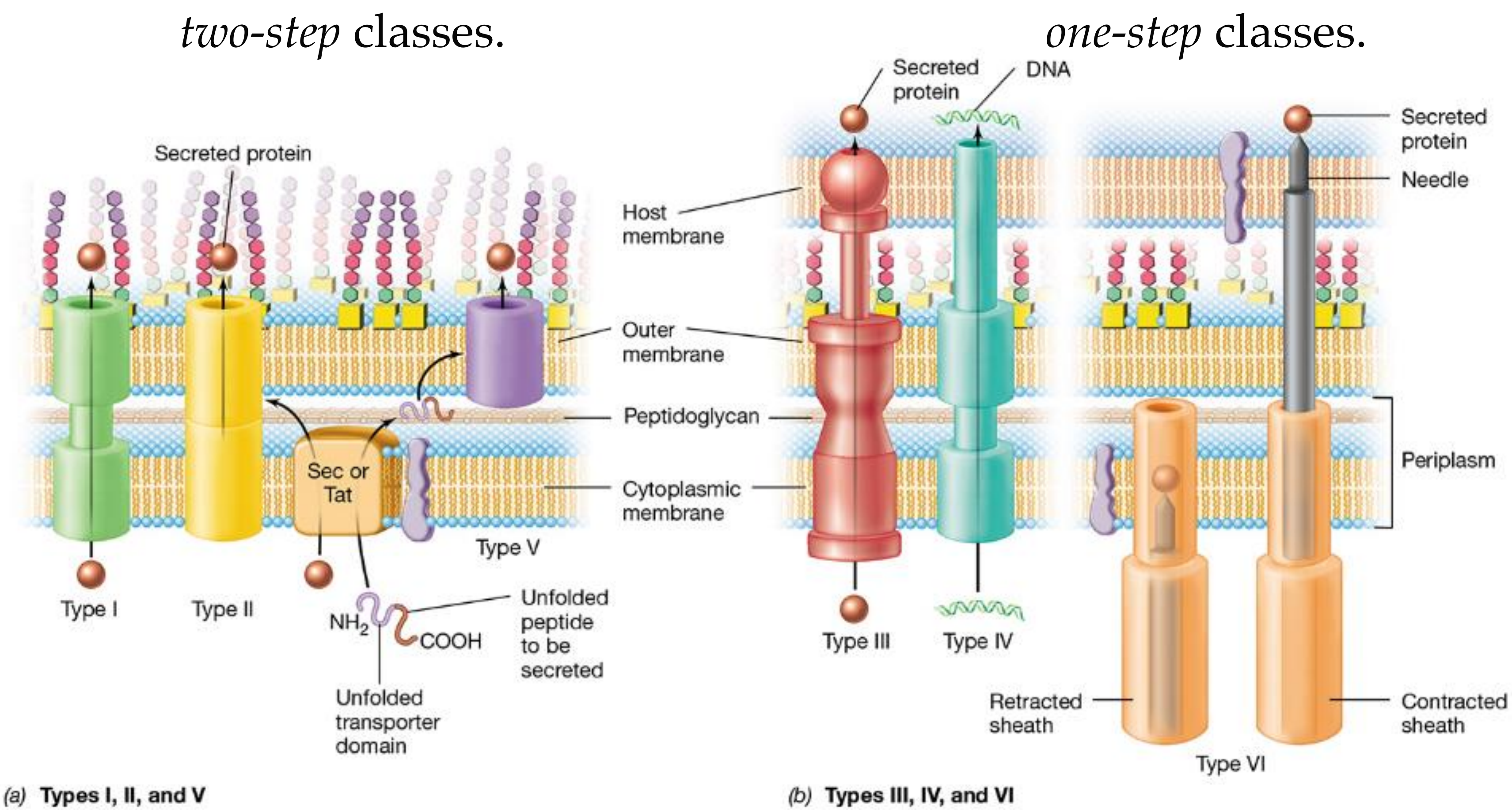

Fig 4.42

Protein secretion systems in Gram-negative Bacteria

(a) Two-step classes. Types I, II, and V secrete proteins **outside** of the bacterial cell. Type I secrete proteins in a single step. Types II and V first require the Sec or Tat system to transport the protein to be secreted across the inner membrane.

- Note that during type V secretion, the Sec system first transports the unfolded transporter domain linked to the unfolded secretion protein through the inner membrane.

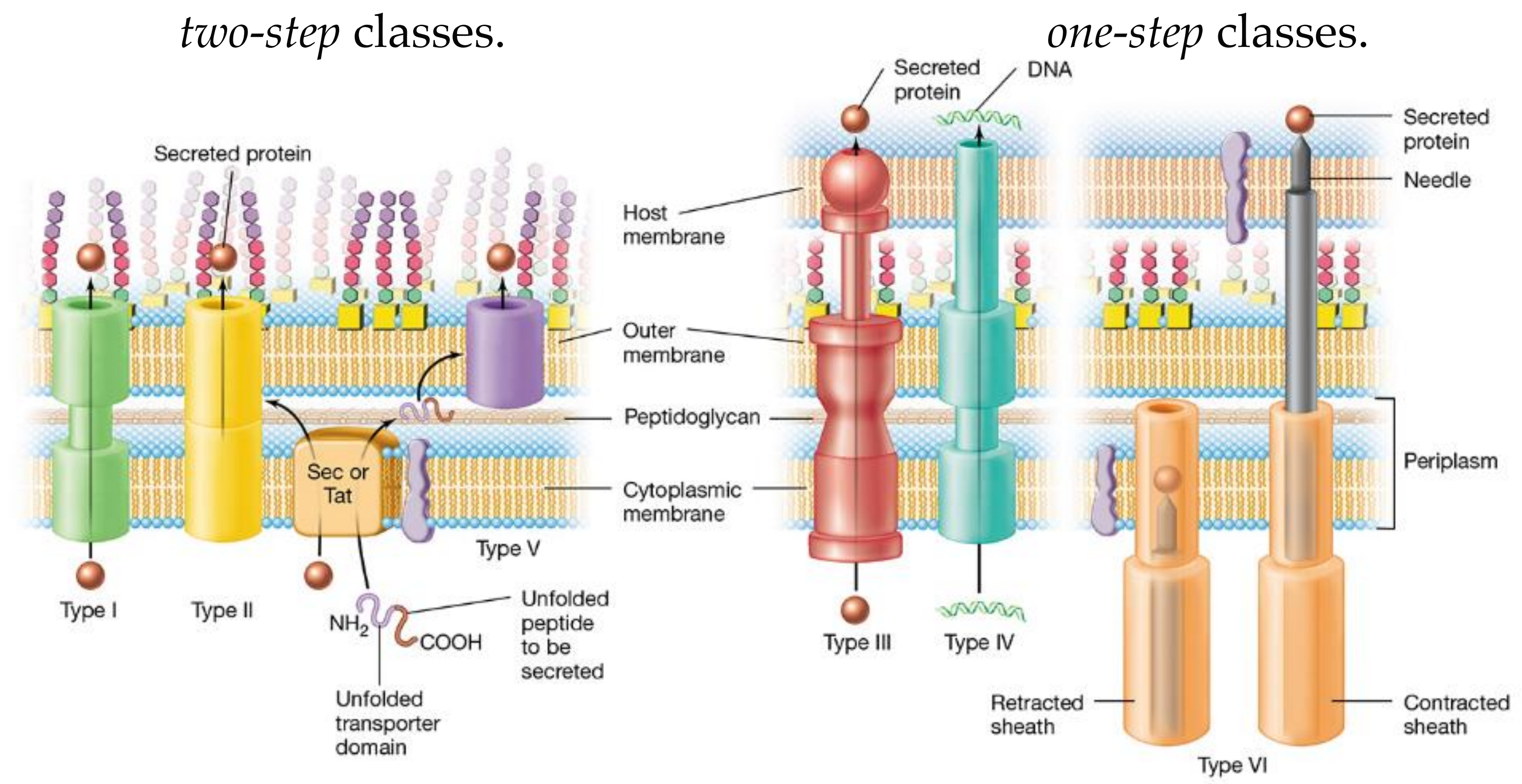

(a) **Types I, II, and V** (b) **Types III, IV, and VI**

Fig 4.42

Protein secretion systems in Gram-negative Bacteria

(b) **One-Step Classes.** Types III, IV, and VI secrete molecules outside of the bacterial cell and into a host cell. Type III systems have been termed the injectisome, while type IV systems are similar to a pilus and also secrete DNA into a host cell. Type VI systems contain a sheath or needle in the cytoplasm that contracts to deliver a protein into a host cell.

Next: Exo-enzymes and their reactions

END OF PROTEIN SECRETION

Hydrolyzing macromolecules and more!

EXOENZYMES

Some exo-enzymes and their polymers

Polymer	Source	Enzymes
**Carbohydrates**		
cellulose	plants	cellulases, glucanases
xylan	plant cell wall	xylanases
starch	plant energy	amylases, phosphorylases
glycogen	animal energy	amylases, phosphorylases
pectin	plants	pectinolytic enzymes
chitin	Fungi, arthropod	chitinases
**Nucleic acids**		
RNA		RNAses
DNA		DNAses
**Proteins**		Proteinases, Peptidases
**Lipids**		Lipases

Microbes often specialize in metabolizing specific molecule types

(e.g., a certain type of polymer)

cellulose (trees)
starch (potatoes)
maltose dextrans (seeds)
pectin (fruit)
chitin (insect and crab shells)
protein (all life forms)
purines (all life forms)

Their food choice depends on their genetic blueprint!

Hydrolytic enzymes can attack at the end (exo) of a molecule or in the middle (endo)!

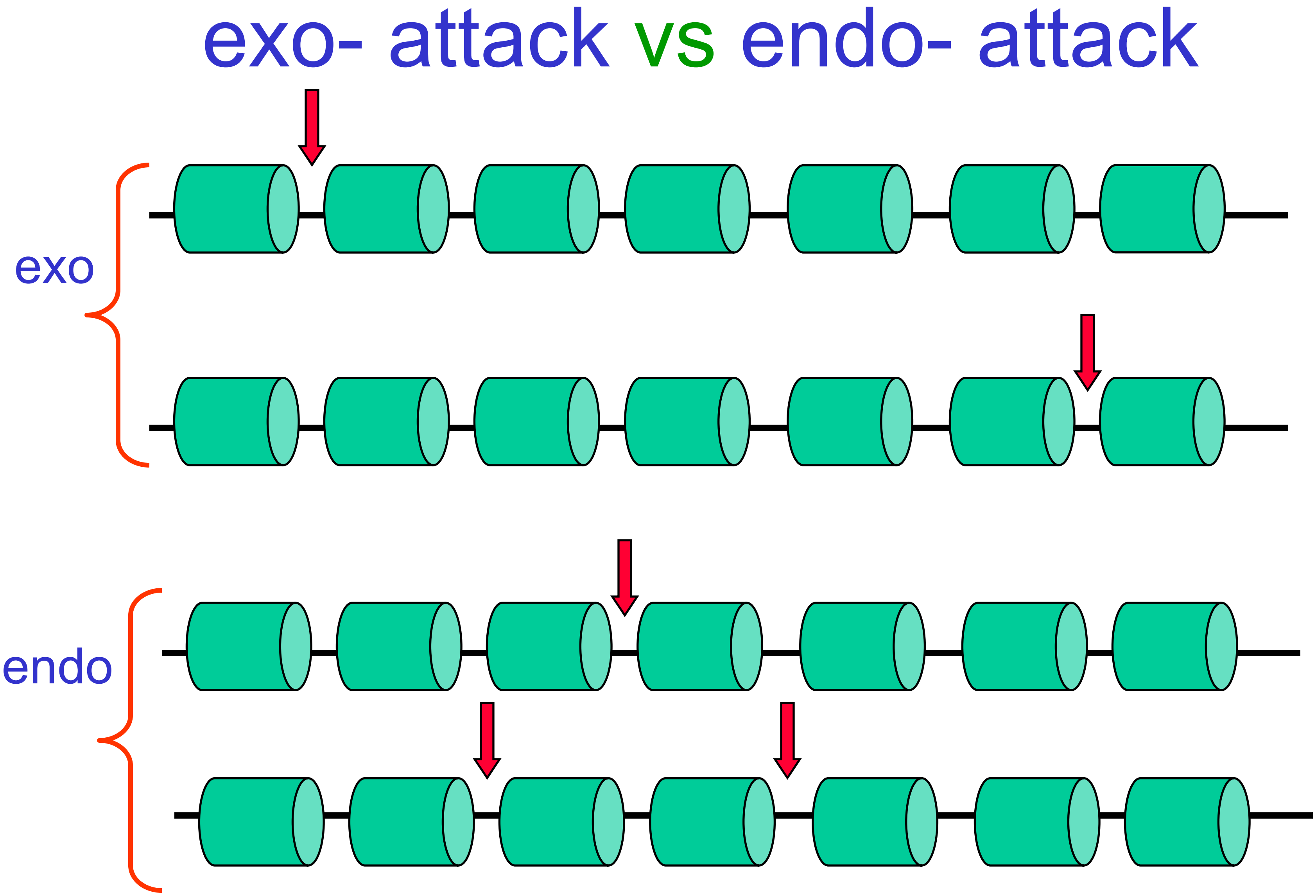

Polymer degradation occurs by:

the hydrolytic attack at bonds joining monomeric units whereby a water molecule is consumed.

the bond energy is usually not conserved!

-this energy is lost as heat.
-this is why your compost heap is always warm!

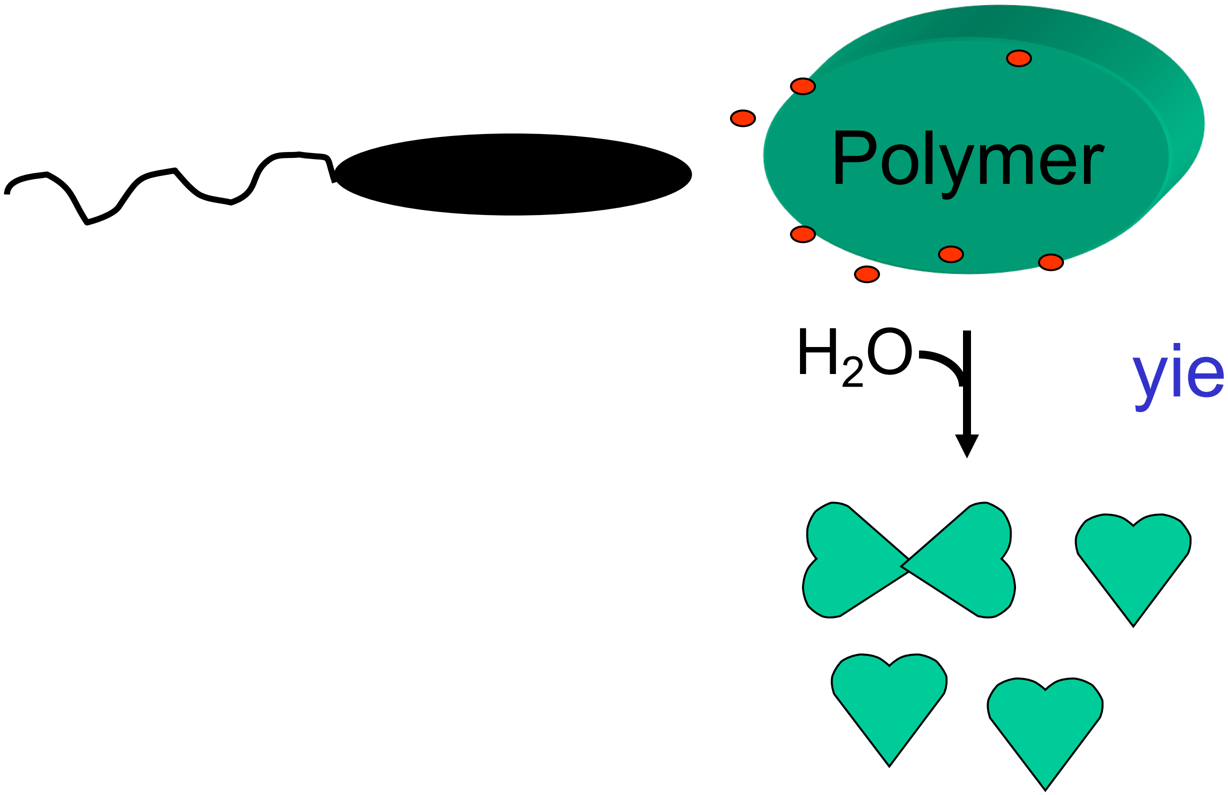

hydrolysis
yields monomers
and dimers

Small molecules are then taken up across the **cell envelope**!

Molecule uptake occurs by active transport systems that a) are usually molecule-specific, and b) require energy input.

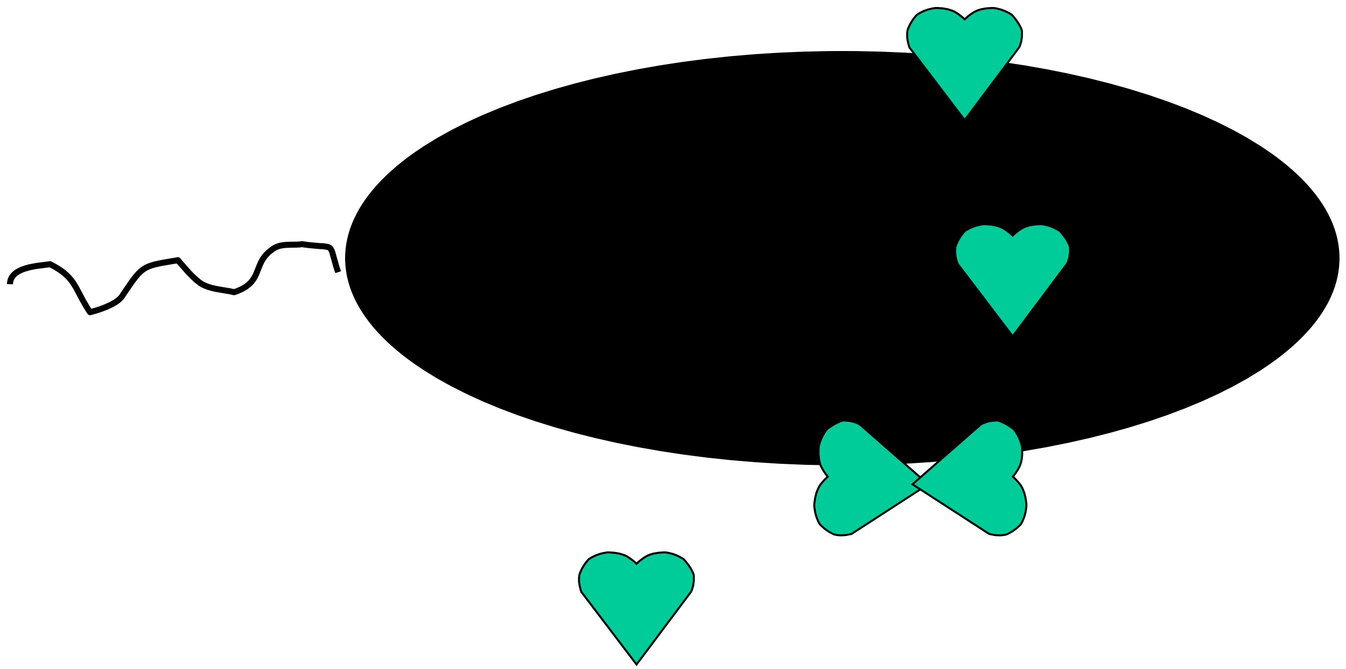

Upon transport into the cell, the molecules are metabolized to harvest energy.

Metabolism includes catabolic and anabolic reactions

Energy Containing macromolecule

- proteins
- nucleic acid
- lipids
- carbohydrates

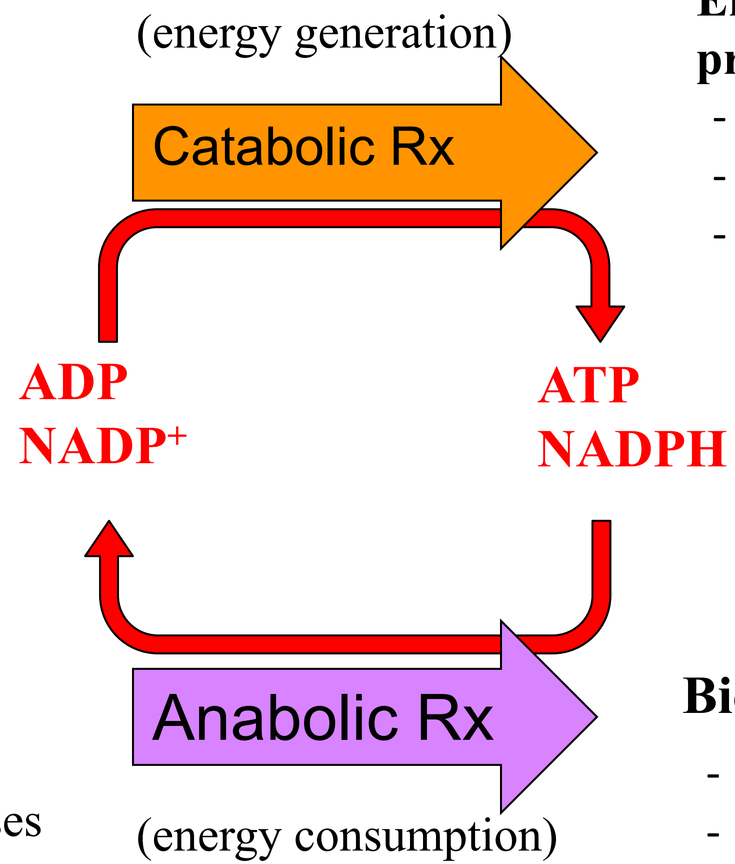

Energy Depleted product

- CO_2
- H_2O
- NH_3

Monomers

- Amino acids
- Nitrogenous bases
- Fatty acids
- sugars

Biomolecules

- proteins
- nucleic acid
- lipids
- carbohydrates

Order of macromolecule breakdown

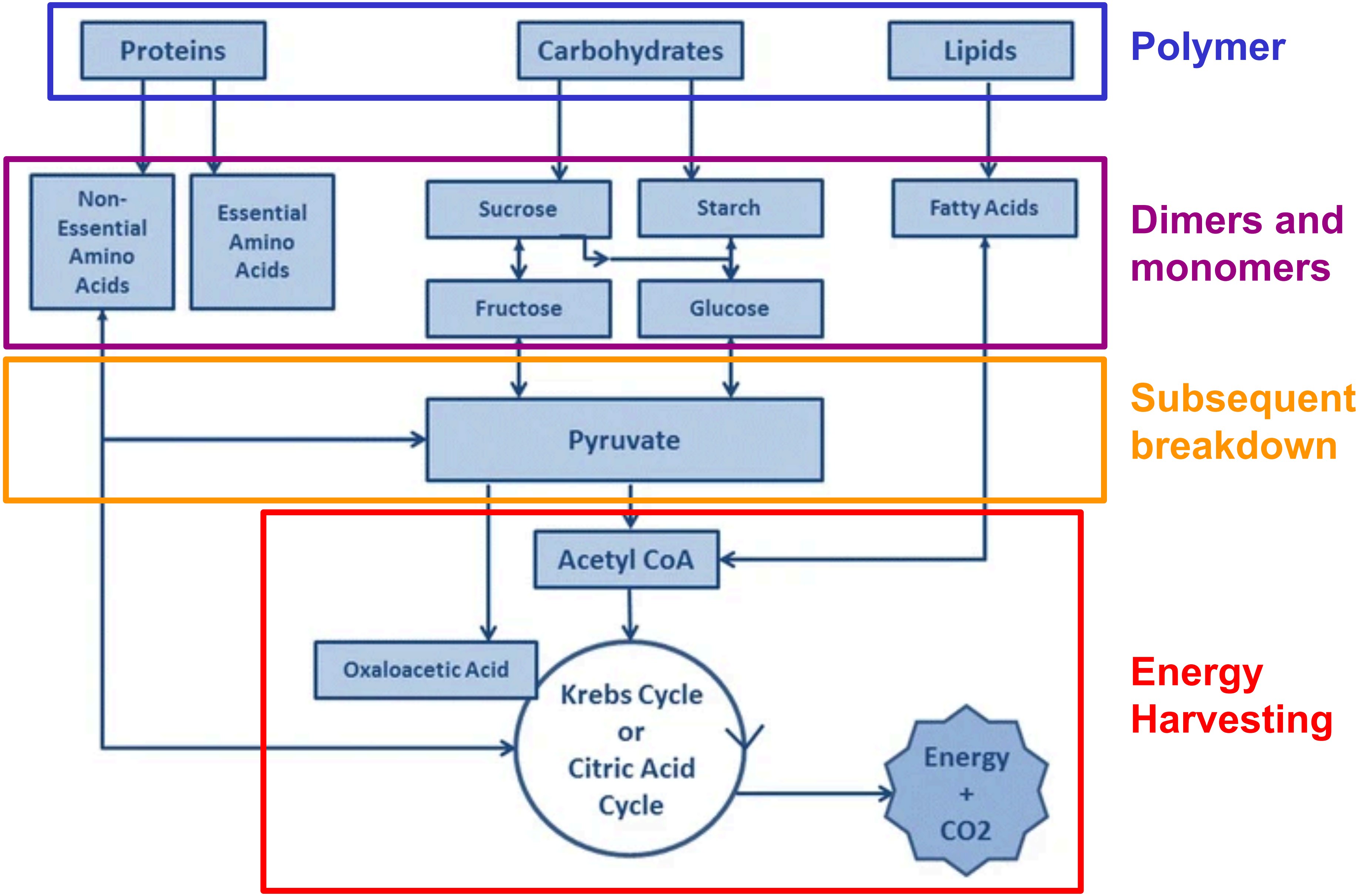

Burns & Trauma **volume 5**, Number: 11 (2017)

Location of macromolecule breakdown

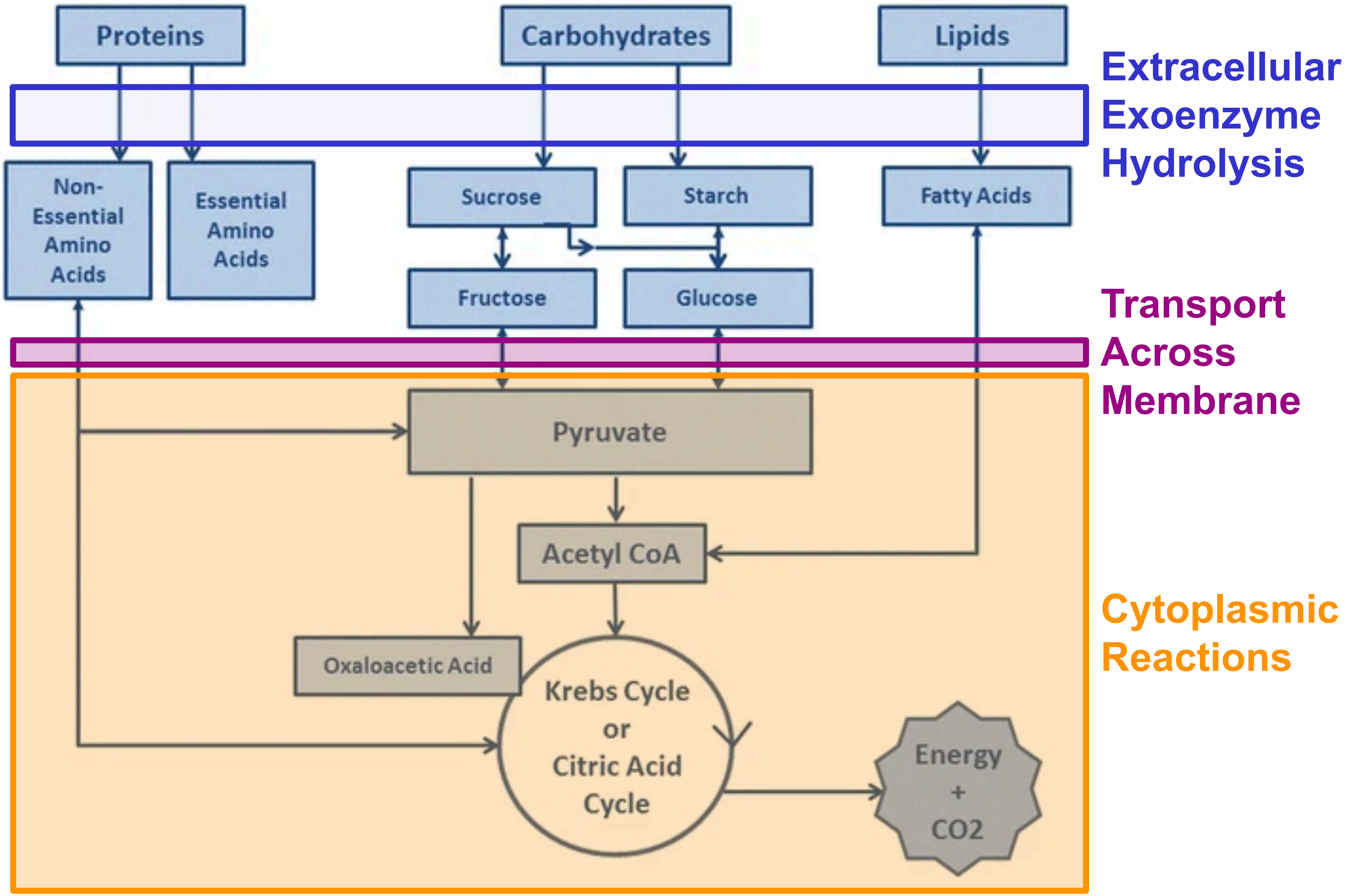

Burns & Trauma **volume 5**, Number: 11 (2017)

END OF EXO-ENZYMES

Prepping monomer molecules for metabolic reactions.

CONVERTER ENZYMES

After transport, some molecules must first be modified before they can be metabolized

"converter" enzymes are synthesized:

they process precursor molecules so the products can then enter central metabolic pathways:

for example...

- Decarboxylation
  - removes a carboxyl group and releases CO_2
- Dehydrogenation (oxidation)
  - removal of hydrogen from an organic molecule
- Deamination
  - removal of an amino group and releases NH_3
- Dechlorination
  - Removal of Cl^-
- “ring activation”

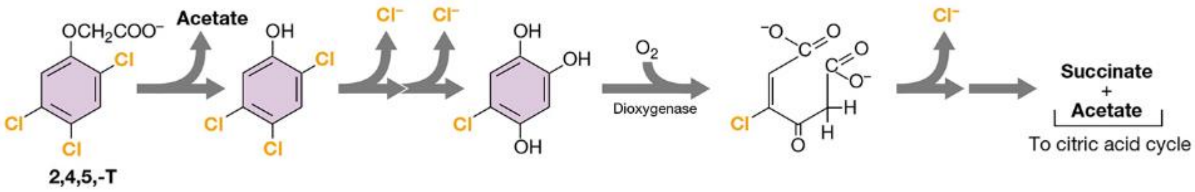

Fig. 22.11; biodegradation of the herbicide 2,4,5-T.

Examples of deamination in *H. pylori*:

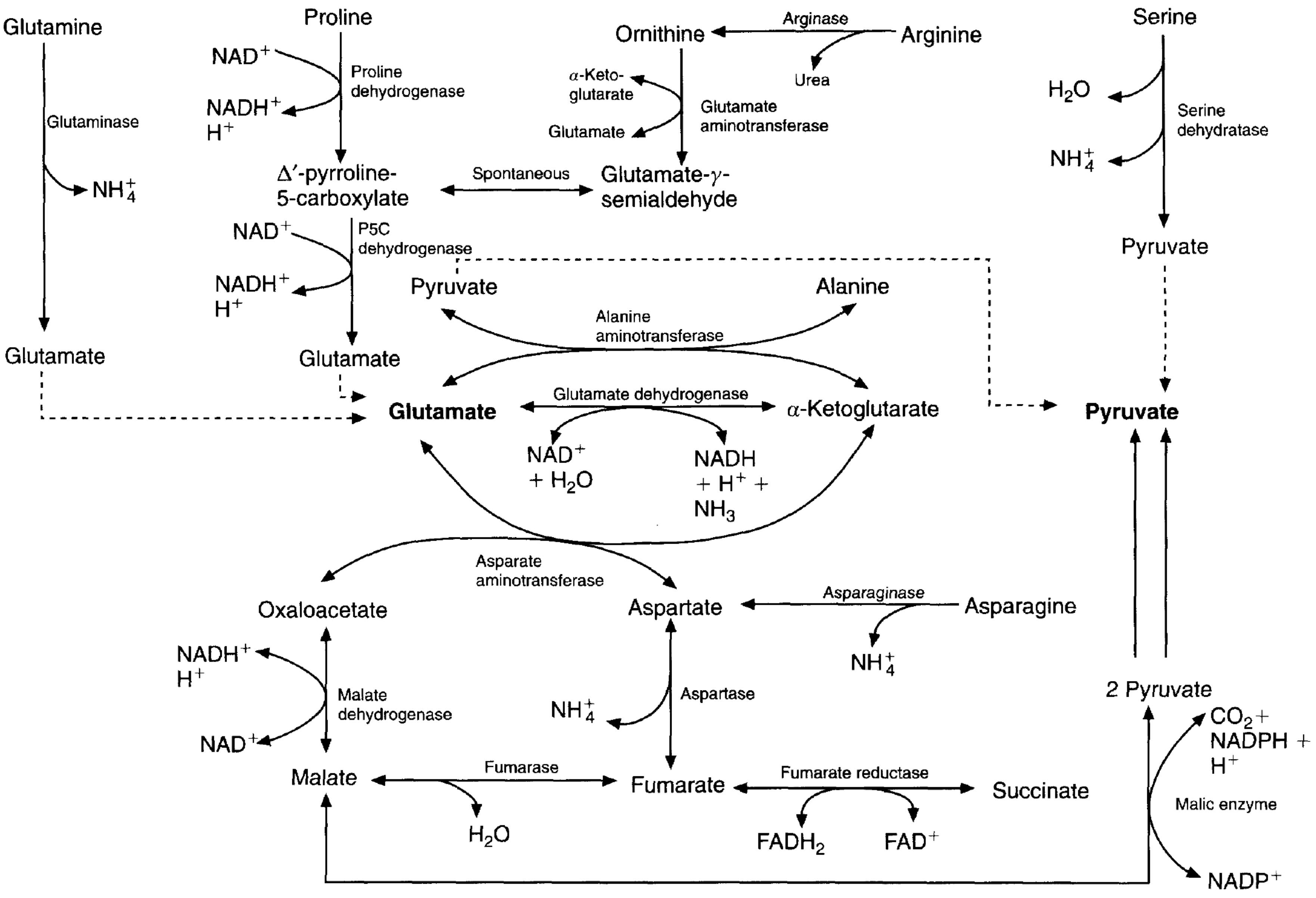

J. Med. Microbiol. - Vol. 46 (1997), 793-800

example of de-hydrogenation:

lactate → pyruvate + NADH + H^+

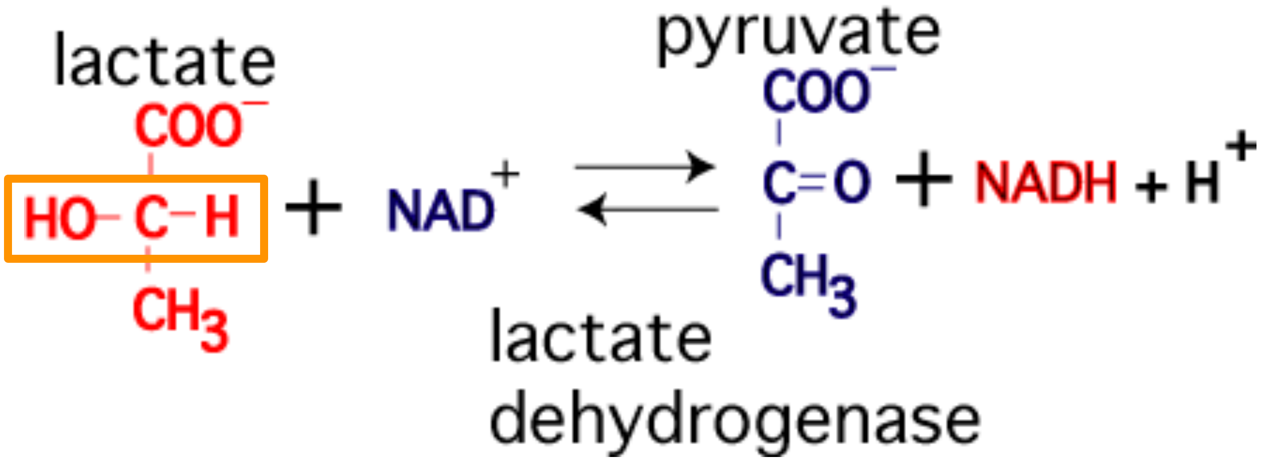

Phospholipases breakdown lipids

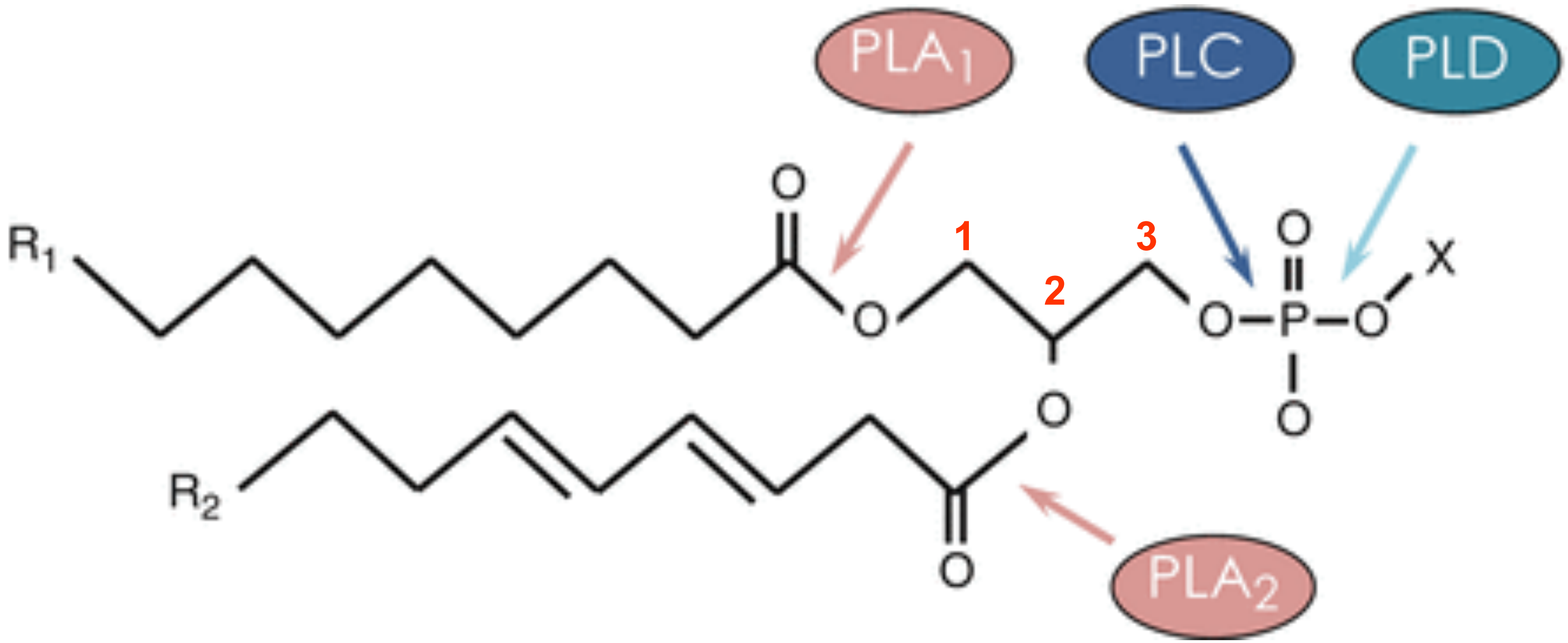

- Phospholipids are composed of a **glycerol-3-phosphate** esterified at the 1- and 2-positions to nonpolar fatty acids (R1 and R2, respectively) and at the phosphoryl group to a polar head group, X.
- **Phospholipase A1 and A2** cleave the acyl ester bonds at position 1- and 2-, respectively. **Phospholipase C** cleaves the glycerophosphate bond, whereas **phospholipase D** removes the head group, X.
- PLA, phospholipase A; PLC, phospholipase C; PLD, phospholipase D

Yang Y.R., Jang HJ., Ryu S.H., Suh PG. (2014) Phospholipases in Health and Disease. In: Tappia P., Dhalla N. (eds) Phospholipases in Health and Disease. Advances in Biochemistry in Health and Disease, vol 10. Springer, New York, NY

Fate of the fatty acid products

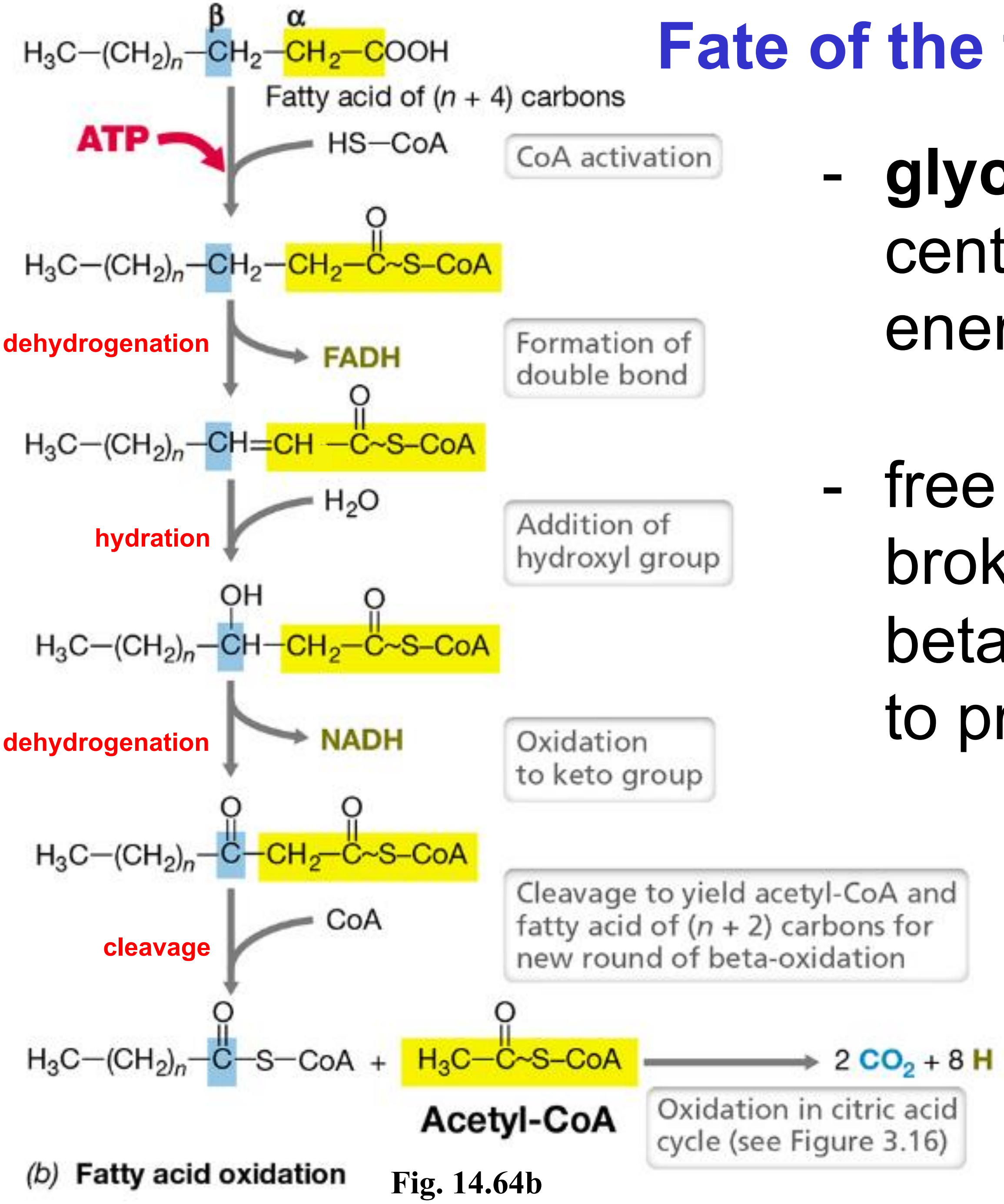

Fig. 14.64b

- **glycerol** is recycled into central biosynthetic or energy pathways.

- free fatty acids are broken down by the beta-oxidation pathway to produce **acetyl-CoA**.

Large oil spill bioremediation by Hydrocarbon-oxidizing bacteria

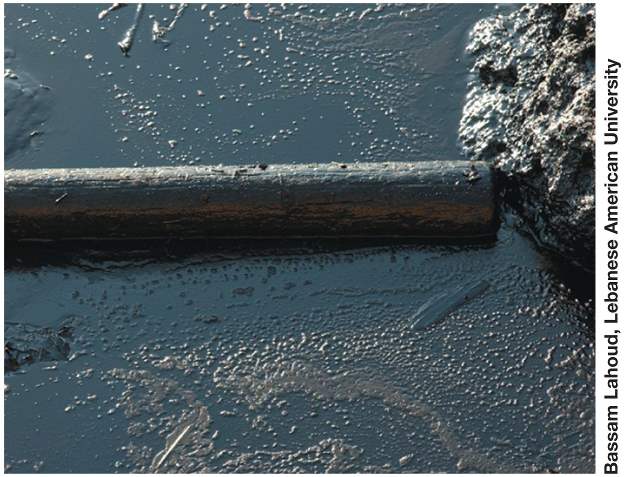

(c) Fig. 22.7c

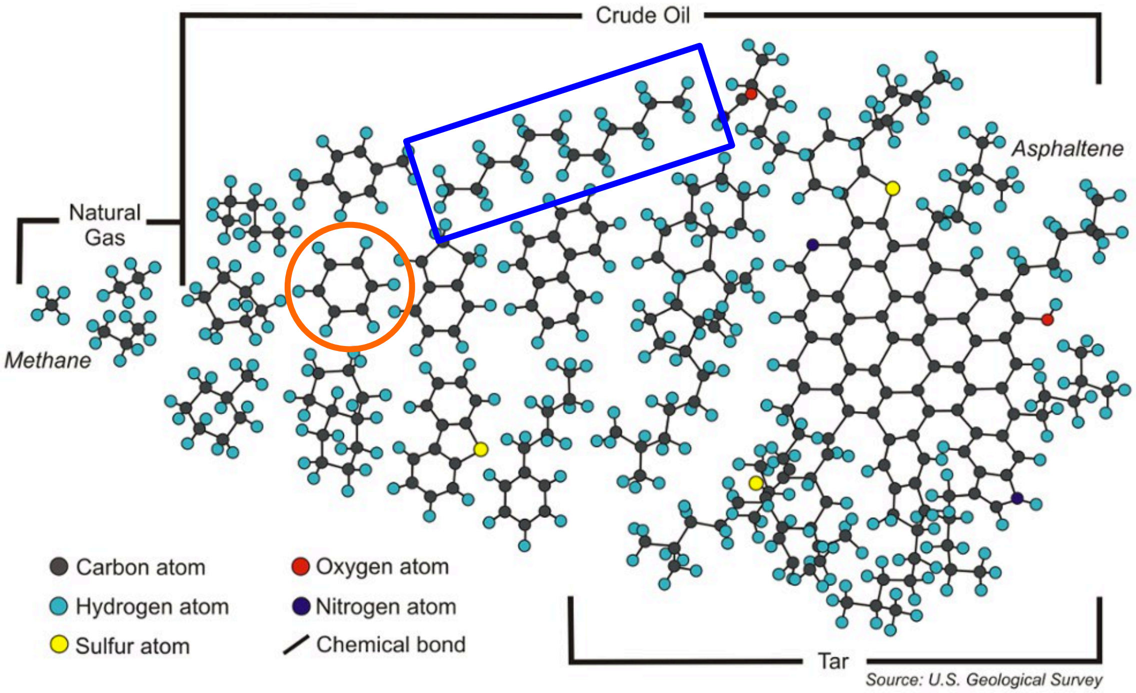

Petroleum contains alkanes in different sizes, including pentane (C_5H_{12}) and octane (C_8H_{18}) to hexadecane ($C_{16}H_{34}$) and alkanes with more than 16C. At the heavier end, paraffin wax has 25C, while asphalt has 35C and up.

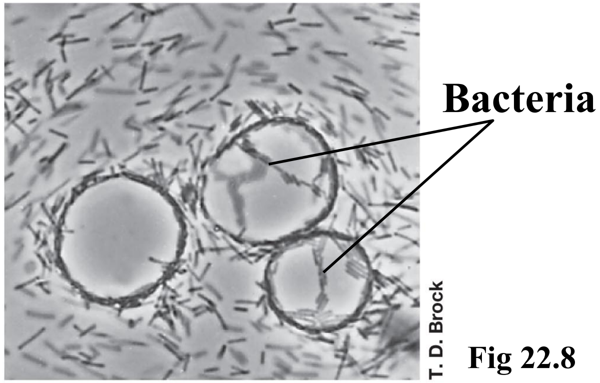

Fig 22.8

Bacteria can break down these hydrocarbons in the presence of O_2, using enzymes called Oxygenases

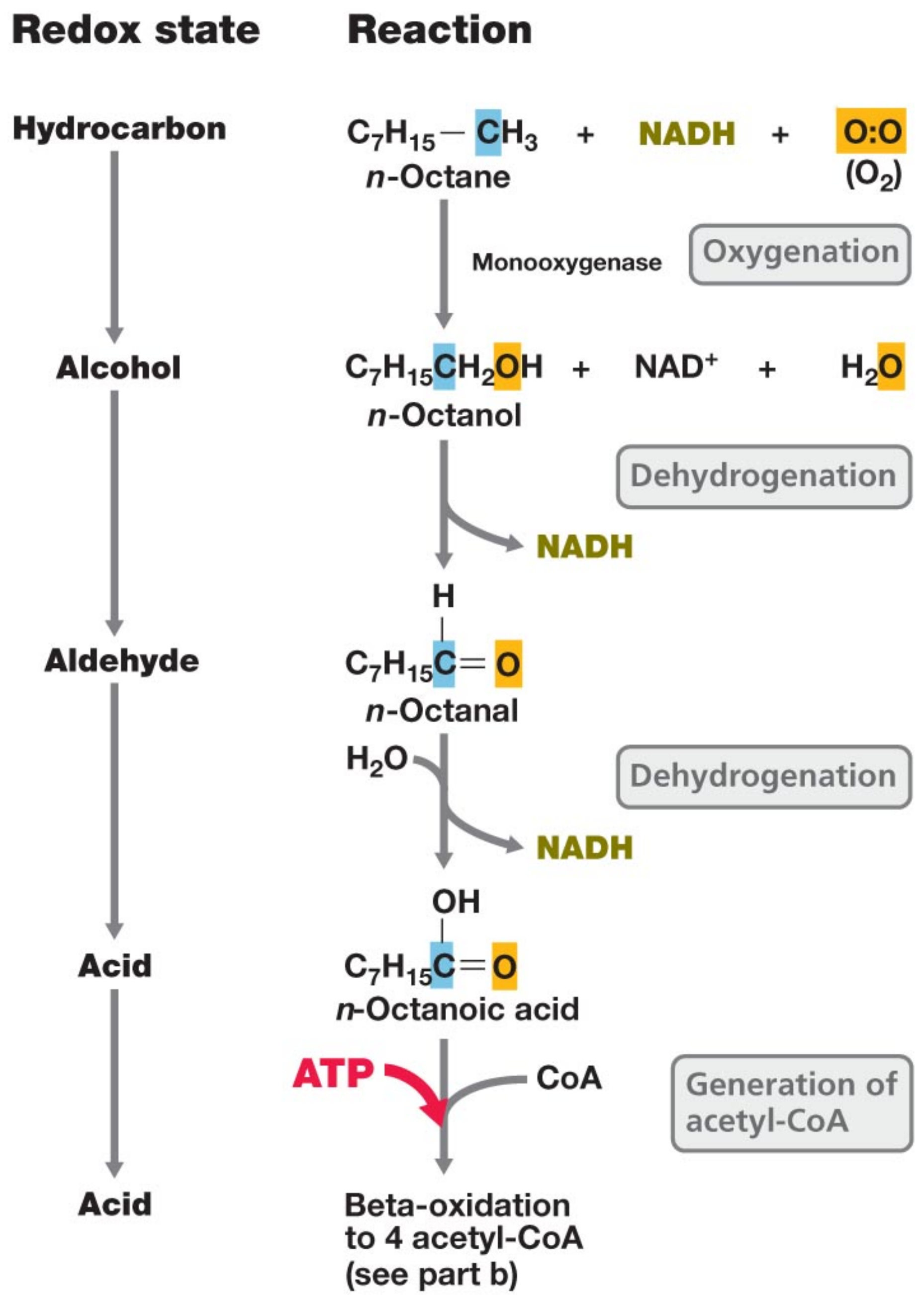

(a) Hydrocarbon oxidation

Fig 14.64a

Monooxygenase reactions add oxygen atoms to hydrocarbons

- Oxygenation of a hydrocarbon creates an alcohol.
- Following a dehydrogenation reaction and NADH production, an aldehyde is produced.
- Another dehydrogenation step, including production of NADH, creates an acid
- This acid can now be oxidized into acetyl-CoA by Beta-oxidation.
    - This is the same reaction used by lipids.

Oxygenases mediate the linearization of aromatic molecules

There are two classes of oxygenases: Monooxygenases which add only one O atom, and dioxygenases which add 2 O atoms.

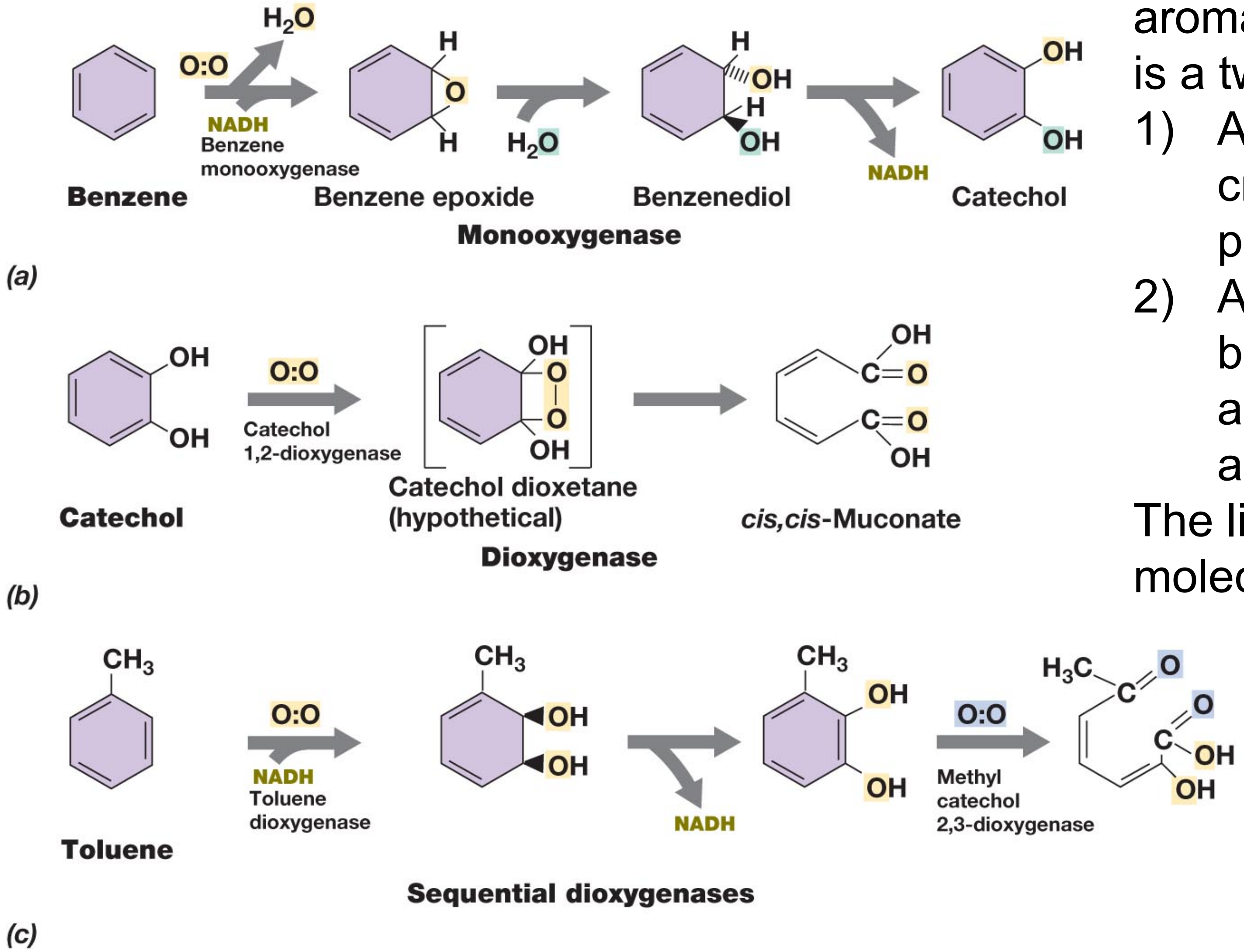

Degradation of an aromatic hydrocarbon is a two step process:

1) A monooxygenase creates a catechol product.
2) A dioxygenase breaks down the aromatic ring into a 'linear acid.

The linearized molecule can now be oxidized by beta-oxidation

Fig. 14.65

Contamination by Xenobiotic aromatic hydrocarbons

Contamination by xenobiotic aromatic hydrocarbons can be bioremediated by the presence of microorganisms that can break down the aromatic rings, using monooxygenase and dioxygenase enzymes.

The process involves “cometabolism”, a process where degradation of the xenobiotic molecule only happens when other organic material is present that can be used as an energy source

Chlorinated compounds are usually quite resistance to microbial degradation and could persist for years.

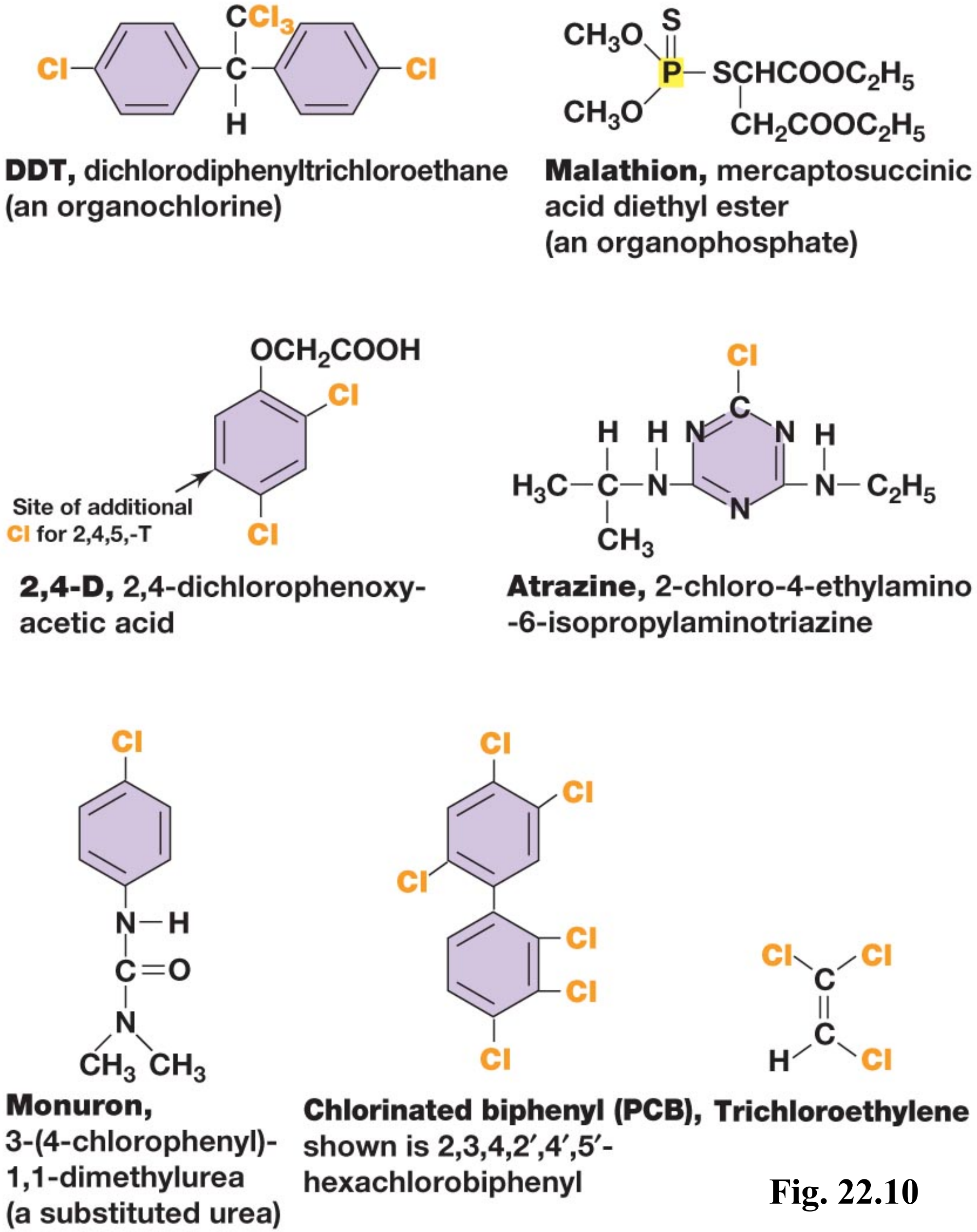

Fig. 22.10

Flow of Carbon:

complex molecules

hydrolysis ↓ no energy gain

monomers

uptake ↓ energy input

conversion maybe energy gain!

pyruvate, acetate

conversion ↓ energy gain!

CO_2 or fermentation products

excretion no energy gain

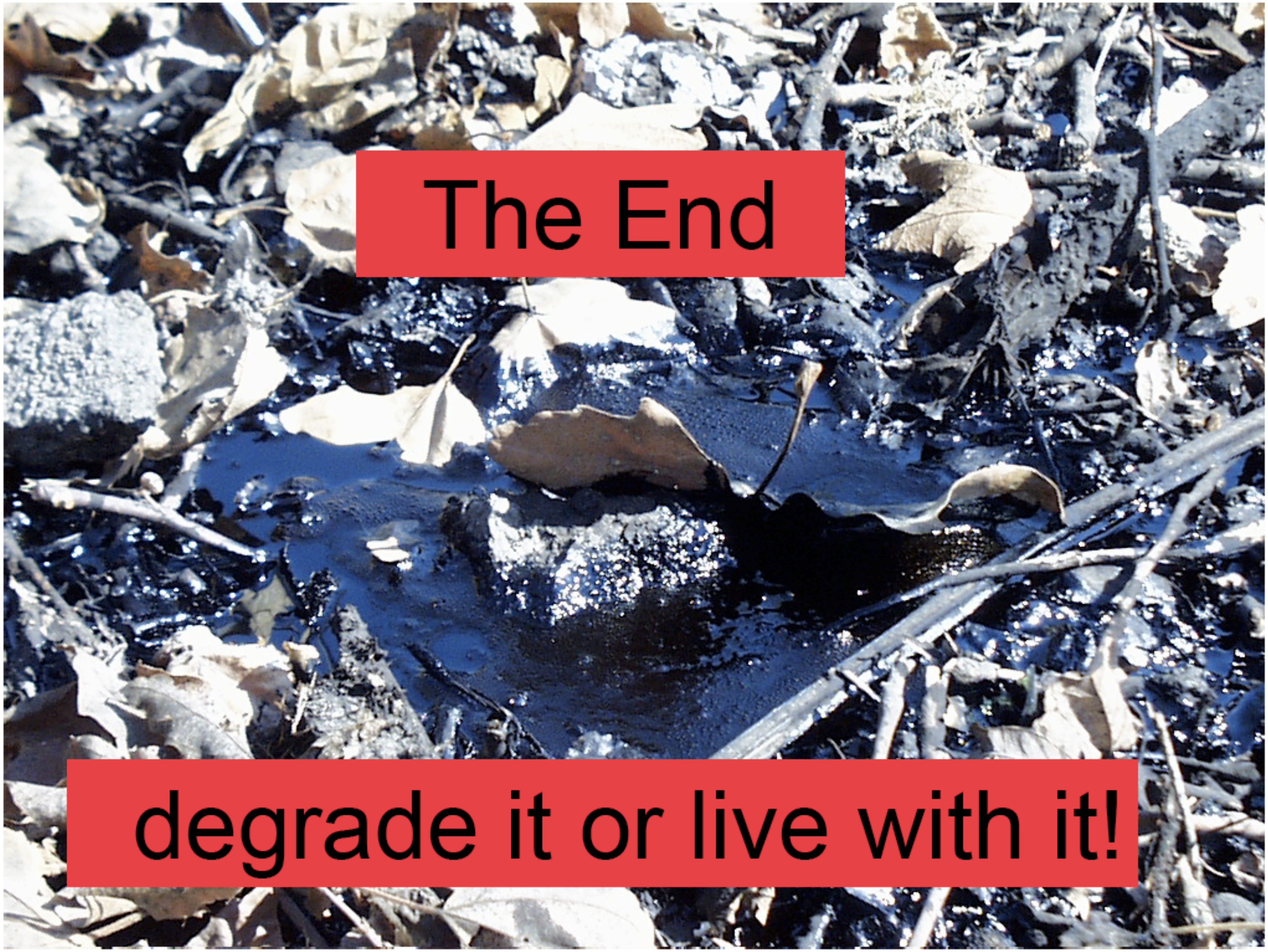

Courtesy of UCLA
