# Supplementary material for: Tackling Real-World Environmental Paper Pollution: A Problem-Based Microbiology Lesson About Carbon Assimilation
Source: Front Microbiol. 2020 Nov 5;11:588918. doi: 10.3389/fmicb.2020.588918 (PMC7674769; doi:10.3389/fmicb.2020.588918)
Supplement: Supplementary file 3 [file Data_Sheet_3.PDF]

Session 12 Learning Outcomes

1. Given the biotic and abiotic sources of carbon and carbon-containing compounds, illustrate the biological flow of carbon, starting from an initial, complex carbon-containing molecule to a final product (CO_2 or fermentation product).
2. Illustrate the enzymatic reactions that hydrolyze carbohydrates, nucleic acids, lipids, and proteins, and identify the various exoenzymes involved in these processes.
3. Identify how different microbes secrete important proteins.

Many recyclable papers end up in the landfill

PRESS PLAY WITH MADELEINE BRAND

The China Ban: Why more of your recyclables are going to the landfill

Written by Jenny Hamel • Aug. 09, 2018 ENVIRONMENT

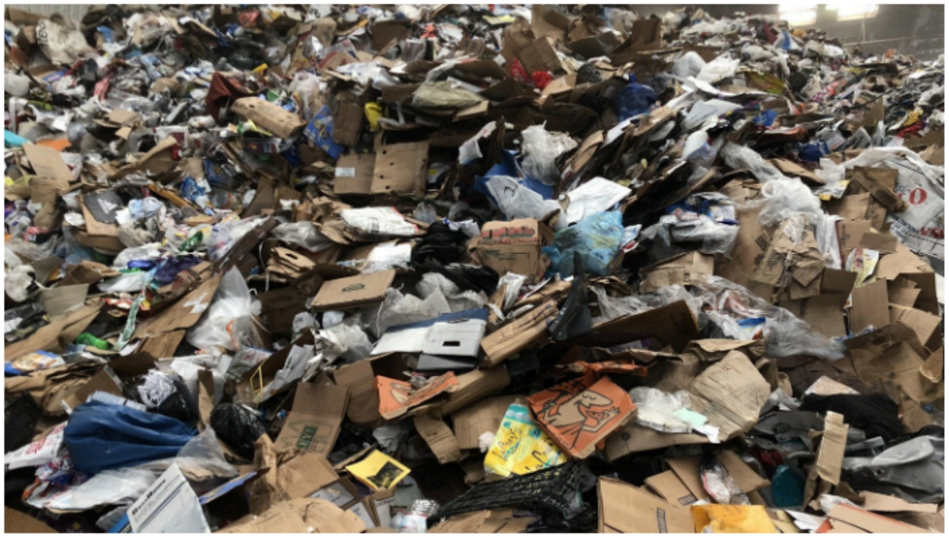

https://www.kcrw.com/news/shows/press-play-with-madeleine-brand/the-china-ban-why-more-of-your-recyclables-are-going-to-the-landfill

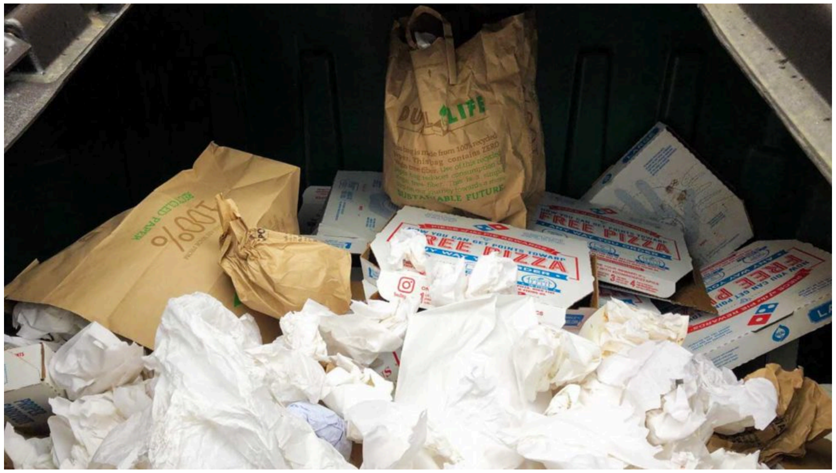

- Since 2001, the recycling market was driven by China, which was buying a lot of what was coming out of our blue recycle bins in the United States.
  - In the process, China was taking half the planet's recycling, so it essentially inherited half the planet's trash and pollution.
- In January 2018, a new Chinese policy called "National Sword" went into effect, banning 24 types of "foreign waste" from entering the country – that includes a lot of plastics and mixed paper, which Americans are accustomed to recycling.
- China also implemented a tough new contamination standard. **Essentially, if any given bale of recyclables is less than 99.5% clean, they reject it.**
  - India announces 1% contamination limit on mixed paper imports to combat heavily contaminated loads (1/14/2020).
- **What type of paper products would be rejected under the National Sword policy?**

Cardboard and paper waste are major challenges in waste management

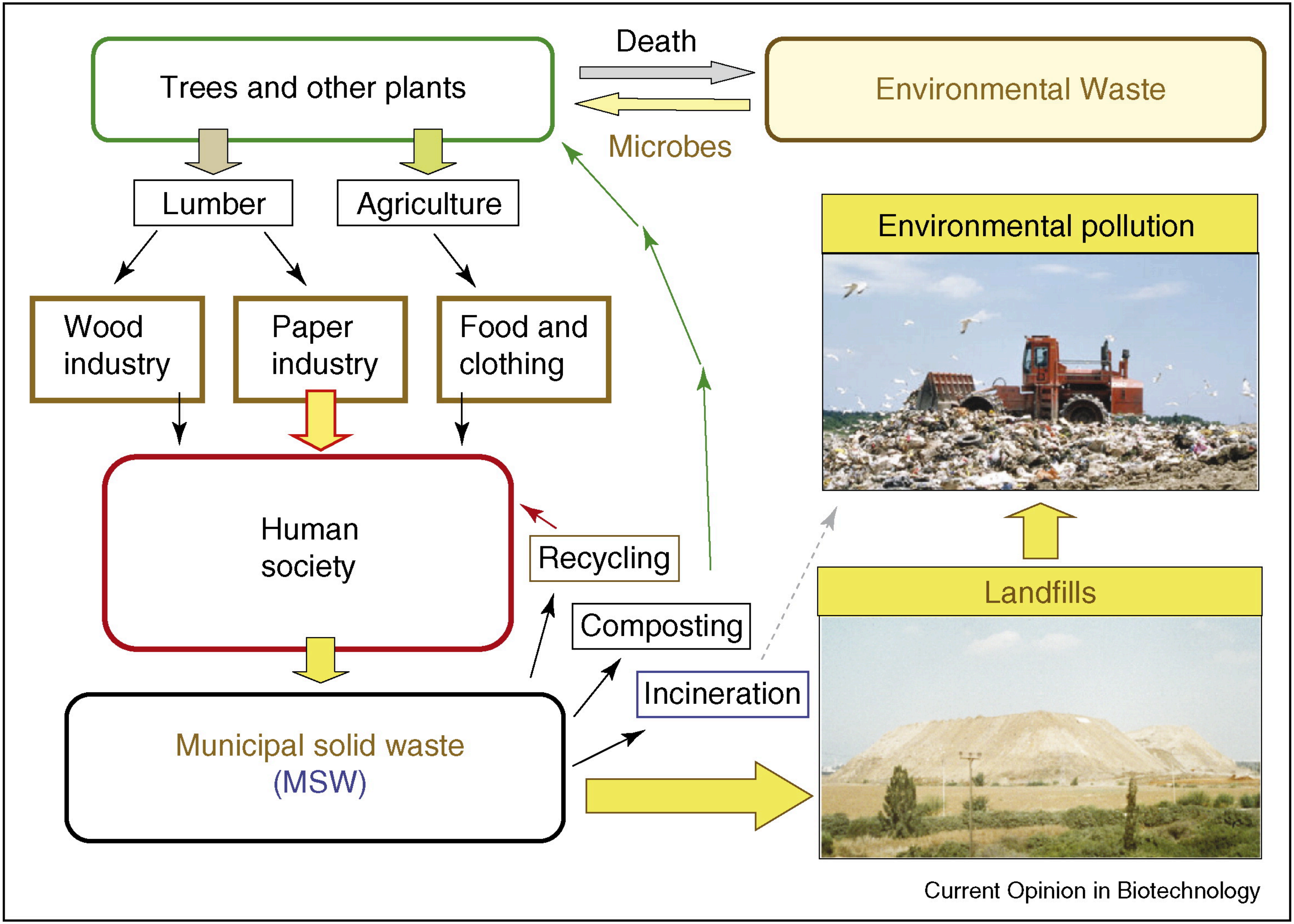

Current Opinion in Biotechnology, Volume 18, Issue 3, 2007, 237–245

Before today, to what extend did you understand the role played by microbes in the biodegradation of paper waste products?

A. Completely understand
B. Understand a lot
C. Somewhat understand
D. Understand a little
E. Do not understand at all

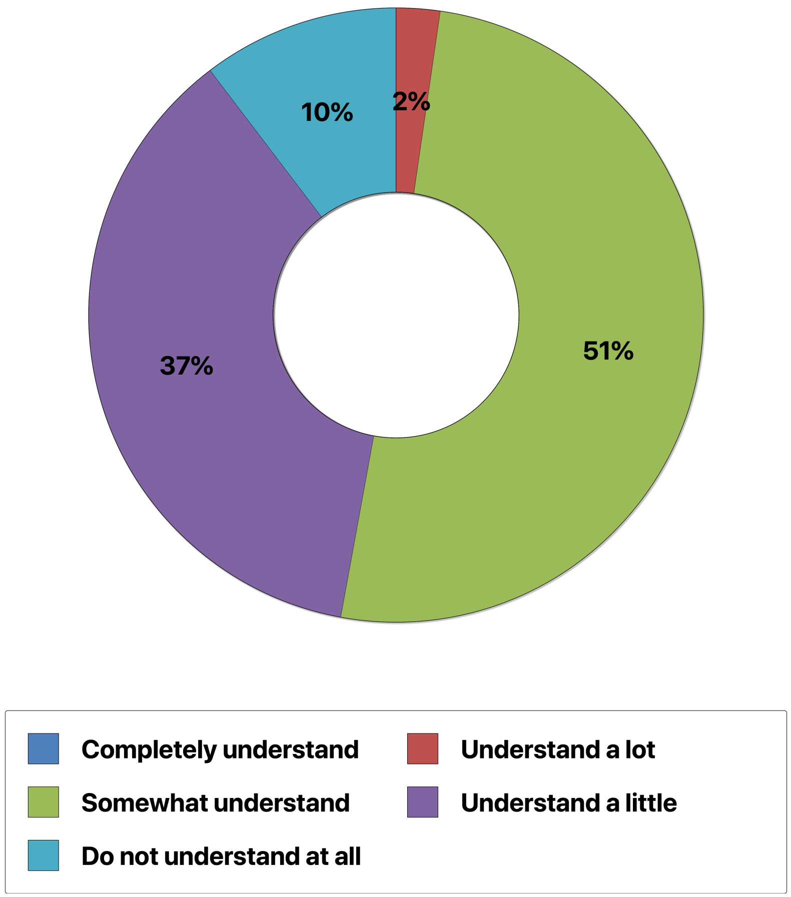

Prediction: paper in the landfill…

A. It remains in the land field, as paper is not degradable.
B. It will decompose primarily by microbial activity involving respiration.
C. It will decompose primarily by microbial activity involving fermentation.
D. Something else will happen…

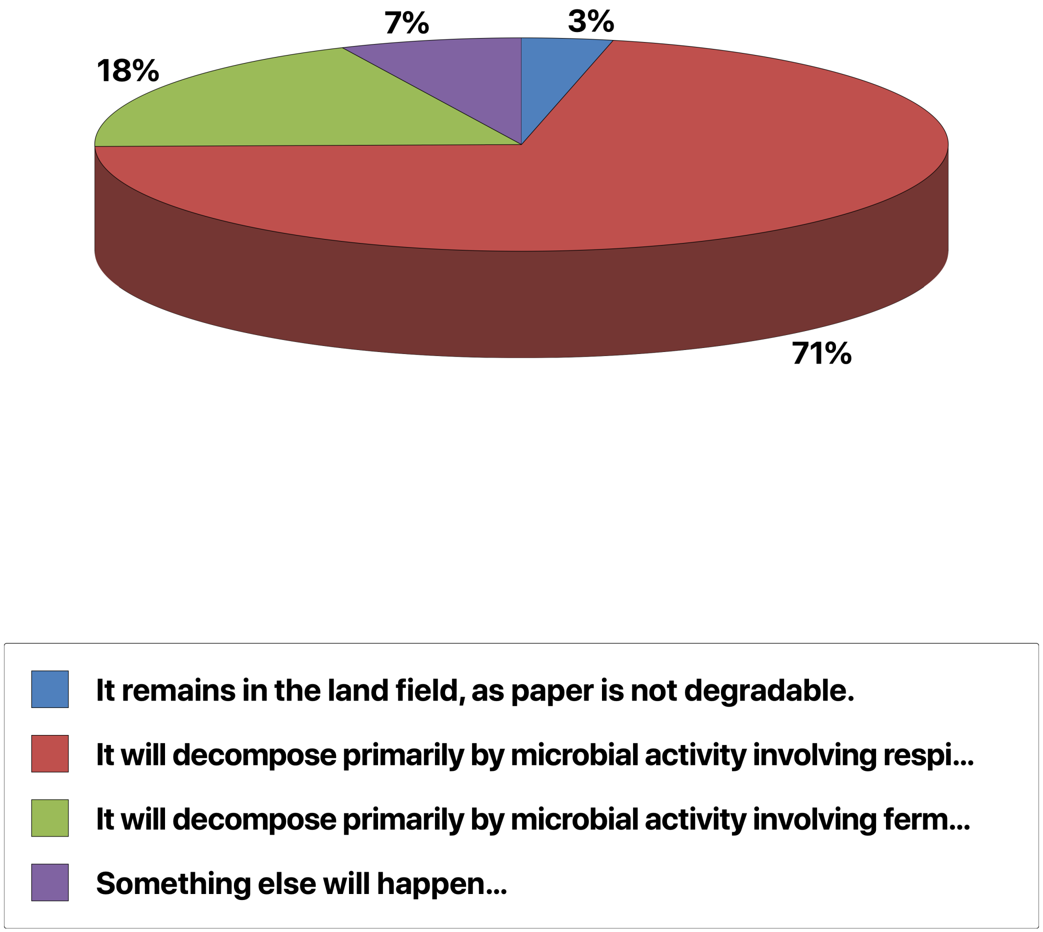

Group exercise, Part 1

Left side of the table

- What is the molecular composition of cardboard and paper?
- Which microorganisms would degrade cardboard and paper?
  - Are these biochemical processes happening aerobically or anaerobically?
- Which exoenzymes would these organisms use?

Right Side of the table

- How would these exoenzymes be secreted?
- How would the products of the exoenzymatic reactions be transported into the cytoplasm of the bacteria?
- Once in the cytoplasm, what biochemical processes would be used in catabolic reactions?

Group exercise, Part 2, Illustrate the process!

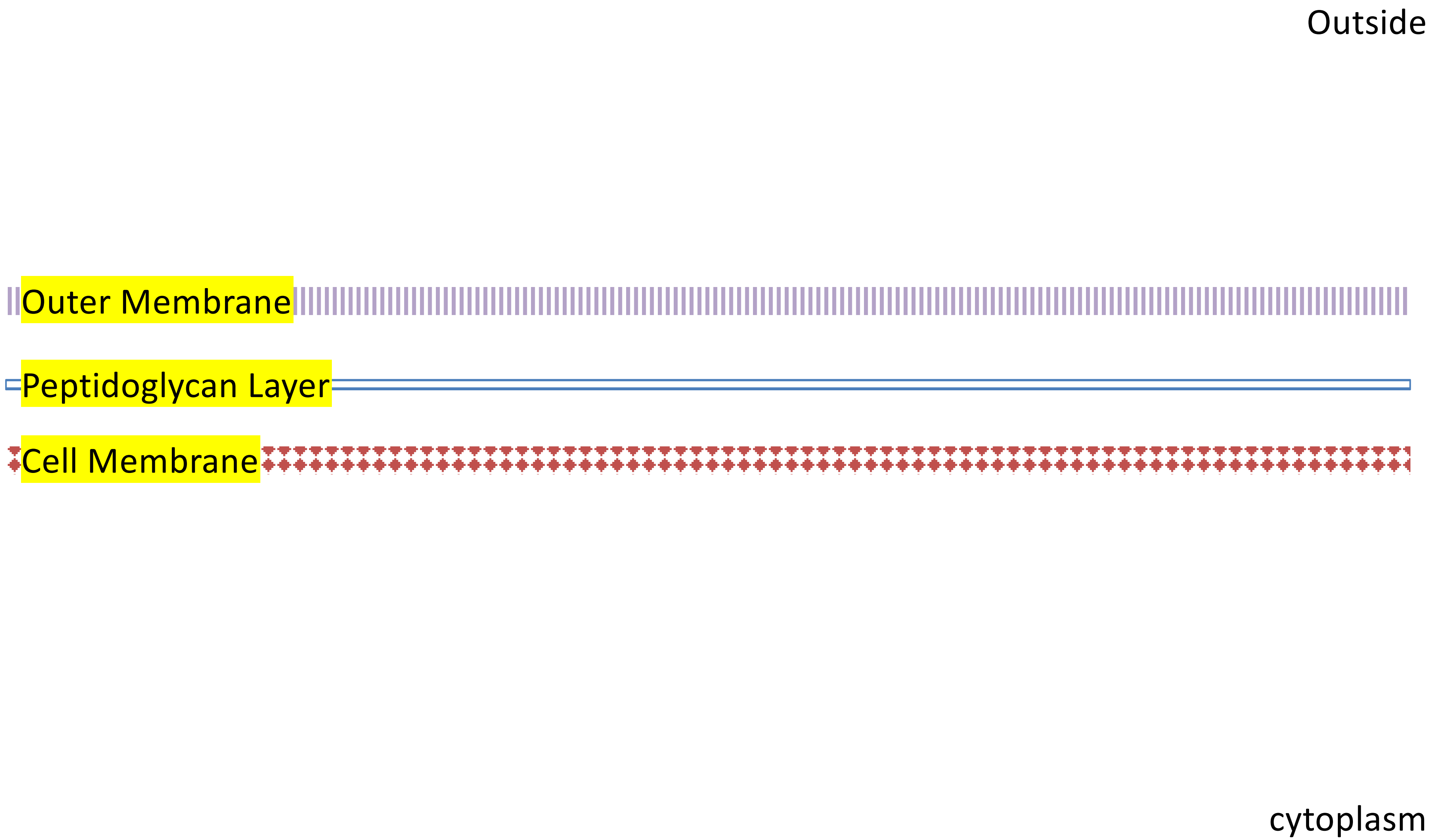

Prediction: paper in the landfill…

A. It remains in the land field, as paper is not degradable.
B. It will suffer water damage.
C. It will decompose by microbial activity involving respiration.
D. It will decompose by microbial activity involving fermentation.
E. Something else will happen…
